# Cyber-resilient machine learning framework for accurate individual load forecasting and anomaly detection in smart grids

**DOI:** 10.1038/s41598-025-31007-z

**Published:** 2025-12-18

**Authors:** M. Tayseer, M. Talaat, Amr A. Zamel, Bishoy E. Sedhom, M. Elgamal, Tomonobu Senjyu, Dongran Song, Islam M. Ibrahim, M. H. Elkholy

**Affiliations:** 1 Electrical Power and Machines Department, Faculty of Engineering, Zagazig University, P.O. 44519, Zagazig, Egypt; 2 Computer and Systems Engineering Department, Faculty of Engineering, Zagazig University, P.O. 44519, Zagazig, Egypt; 3 Electrical Engineering Department, Faculty of Engineering, Mansoura University, Al Mansurah, Egypt; 4 Department of Electrical and Electronics Engineering, University of the Ryukyus, Okinawa, 903-0213 Japan; 5 School of Automation, Central South University, Changsha, 410083 China; 6 Faculty of Engineering and Technology, Egyptian Chinese University, P.O. 11787, Cairo, Egypt; 7 Interdisciplinary Research Center for Smart Mobility and Logistics, King Fahd University of Petroleum and Minerals, Dhahran, 31261 Saudi Arabia; 8 Computer Engineering Department, Engineering and Information Technology College, Buraydah Private Colleges, Buraydah, 51418 Saudi Arabia

**Keywords:** Smart meters, Load forecasting, K-MEANS clustering and neural networks (KMEANS–NN), Anomaly detection scheme (ADS), Cyber-attack scenarios, Resilient support vector machine, Energy science and technology, Engineering, Mathematics and computing

## Abstract

With the evolution of smart grids, accurate and secure predictions of the electricity load become crucial for efficient energy management and reliability. In this paper, a scalable and cyber-resilient methodology for electricity consumption forecasting on individual smart meter level based on machine learning and anomaly detection schemes is proposed. The proposed technique utilizes K-MEANS Clustering and Neural Networks (KMEANS–NN) to enhance Individual Load Forecasting (ILF) with reduced computational complexity and high prediction accuracy. A Principal Component Analysis based One-Class Support Vector Machine (PCA–OCSVM) model is employed as an Anomaly Detection Scheme (ADS) to identify the false data injection attacks in smart meter telemetry. The system uses five months of real-world data from $\:\text{2,089}$ smart meters gathered under the supervision of Electrical Distribution Sector (EDS) of Suez Canal Authority (SCA) in Egypt. KMEANS–NN strategy reduces significantly MAAPE by up to $\:25.6\%$ and cuts computational time from days to minutes. It improves forecasting accuracy across four proposed models: ARIMA, CTREE, MLP and NNETAR. To assess the cyber-security profile, $\:50\%$ of the dataset is orchestrated with scaling, ramping and random cyber-attack simulation. Proposed ADS achieves $\:99.3\%$ overall accuracy, $\:100\%$ sensitivity, $\:98.62\%$ precision, $\:98.6\%$ specificity and F1-score of$\:\:0.9896$, whereas it’s $\:100\%$ accurate on clean data. This integrated model offers accurate, efficient, and secure load forecasting presenting good potential for its deployment in large-scale smart grid environments.

## Introduction

Smart meters act effectively as two-way communication channels correlating the electricity suppliers with customers. In this regard, customers can receive real-time information about their electricity usage enabling them to reduce their overall costs and consumption. Nowadays, the growing adoption of smart communities and distributed energy resources has transformed the conventional consumers into others who can generate, store, and trade electricity within local energy markets^1,2^. This evolution has made secure energy trading platforms where transparent and credible energy transfers between parties are enabled. These platforms usually integrate blockchain, smart contracts, and secure communication protocols to enable transaction integrity, prevent data tampering and support the privacy preservation^3,4^. Also, the electricity suppliers depend mainly on smart meter measurements to forecast the future demand accurately avoiding shortages and surpluses. In this regard, accurate and resilient Load Forecasting (LF) mechanisms are essential for optimizing Peer-to-Peer (P2P) energy trading, setting fair market prices, and managing grid stability^5,6^.

Coping with the increasing digitalization of power systems, blockchain technology has emerged as a vital foundation for decentralized energy transactions in smart grids^7–10^. It provides a tamper-evident and distributed ledger that has a record of all transactions openly and securely, hence rendering a central authority unnecessary. Cryptographic proof and consensus methods employed in blockchain ensure data integrity, traceability, and trust by all participants in local energy markets^11^. This decentralized structure enables P2P energy trading, dynamic pricing, and automated settlement through smart contracts, improving both transparency and operational reliability^12,13^. Furthermore, combining blockchain with machine learning-based forecasting and anomaly detection methods enhances transparency, protects against malicious manipulation, and strengthens cyber-resilience within smart grid ecosystems.

The frequent volatility and expected operational ambiguity of electric load profiles significantly affect the consumption forecasting profile of smart meters especially for residential loads paving the way for a fertile challenge domain. This obstacle makes the smart meter consumption data collected to predict future loads to be categorically vulnerable to cyber-attacks due to vulnerabilities in the communication system. In other words, the rapid digitalization of residential energy infrastructure has increased the susceptibility of smart meters to cyber-attacks in addition to the false data injection attacks. These hazardous attacks cause compromises in smart meter data integrity transferred between consumer premises and utility control centers leading to inaccurate billing, operational disruptions, or the large-scale grid instabilities^14,15^. Therefore in a natural result, these obstacles in turn pose the need for the deployment of the trustworthy intrusion detection mechanisms as part of modern microgrid infrastructures^16,17^. For overcoming these obstacles, numerous intrusion detection models have been proposed, including signature-based, rule-based, and machine learning-based anomaly detection models. Among these, unsupervised learning models such as One-Class Support Vector Machine (OCSVM) and Principal Component Analysis (PCA)-based models have been seen to be particularly well-fitted for identifying unusual patterns of consumption without being dependent on labeled attack data^11,12^. These techniques are very flexible and precise to detect never-before-seen attack vectors, for which reason they are best suited for real-time intrusion detection in residential area smart meter networks. In this context, integrating the cyber-resilient forecasting frameworks with the anomaly detection schemes into these transactional infrastructures is a significant step towards the achievement of intelligent, and sustainable energy management in smart communities^18,19^.

In this manuscript, the main core in the operation of smart meter is its LF capability profile regarding categorical cyber-attack scenarios. LF estimates the preliminary desired power or energy helping electricity suppliers to schedule maintenance, retail sales and execute long-term planning. The utilities depend mainly on LF approach to anticipate future electricity demand to make informed decisions regarding resource allocation, infrastructure investments and operational strategies. The traditional meters poorly respond to the frequent changes of both production and demand profiles of the power system as a result of the expansion in integrating the Renewable Energy Sources (RES) and the development of their innovatively control methods^20^. Smart meters are provided to electricity suppliers as iconic alternatives, attributed to their LF capabilities in balancing intelligently the output generation and the actual demand.

The firm relation between RES and LF of smart meters can be highlighted for Photovoltaic (PV) forecasting profile. The accurate PV forecasting is a crucial element in balancing renewable energy generation with the consumer demand in smart grids^21,22^. However, reliance of PV prediction models on actual measurement and meteorological data makes them vulnerable to cyber-attacks in the forms of intrusions and false-data injection, which may severely degrade forecast quality and grid reliability. Recent studies emphasized the necessity to incorporate data intrusion defense mechanisms into forecasting frameworks directly for the purpose of ensuring predictive accuracy as well as operational security^23,24^. These methods include data validation filters, statistical anomaly detection, and machine-learning-based intrusion detection that can identify aberrant inputs before they propagate through forecasting models^25^. By integrating these defense mechanisms, PV forecasting frameworks become more robust and capable of maintaining reliable predictions even under adversarial data conditions.

The challenge of enhancing LF performance of smart meters is a fertile domain for scientific community to reach a reasonable computational accuracy in order to achieve energy flow balance in the Integrated Energy Systems (IESs) for reliable operation^26^. Energy balance is critical for planning, operating and controlling the IESs as well as for commercial transactions in new energy markets. Considering the State Grid Corporation (SGC) of China, reducing the LF errors by $\:1\%$ could potentially prevent approximately $\:\text{58,000}\:megawatts\:\left(MW\right)$ of power waste annually^26^. Consequently, LF is classified as a crucial component of power system management representing a primary approach in this paper for tracing the main framework structure of proposed studies for reliable smart meter operation.

Arithmetically, LF analysis is divided into two modeling schemes: Aggregated-Level Load Forecasting (ALLF) and Individual Load Forecasting (ILF). This distinction indicates the complicated granularity of LF requirements in modern power systems. ALLF focuses on predicting total electricity demand for a specific area or group of consumers. It is typically performed at transmission level. Conventional machine learning methods such as Linear Regression Method (LRM), Support Vector Regression (SVR), Artificial Neural Networks (ANNs) and the Auto Regressive Integrated Moving Average (ARIMA) are effectively integrated to increase the accuracy of ALLF analysis^27–29^. While ILF aims to estimate the electricity load profile of individual customers or smart meters. It is typically performed at distribution level. Accurately, forecasting the individual load especially residential one at the smart meter poses a significant challenge due to its inherent volatility and uncertainty. Unlike ALLF of a smoother and more predictable pattern, ILF is subjected to frequent fluctuations and disturbances complicating its profile for accurate modeling and prediction. This high level of ILF volatility stems from various factors including the individual household characteristics, occupancy patterns, appliance usage and stochastic events. Therefore, forecasting individual load requires more sophisticated methods to effectively capture the underlying dynamics and complexities of individual consumption patterns^30–32^. ILF accuracy enhancement is related to the use of clustering and integration strategies as promising approaches for overcoming the ambiguity of load profiles^27,31,33–35^.

According to the previous section, the residential consumption patterns need advanced computational and controlling methods to overcome their complicated forecasting profiles, so there are remarkable recent advances in Deep Learning (DL) and Machine Learning (ML) that have been of great promise to predict energy consumption in residential microgrids, where home prosumers exhibit diverse and evolving load patterns^36,37^. Learning-based approaches such as Recurrent Neural Networks (RNNs), Long Short-Term Memory (LSTM) models, Gated Recurrent Units (GRU) and ensemble hybrid structures have been widely utilized to capture nonlinear temporal dependencies in consumption data and to adapt to distributed microgrid operating conditions^38^. These methods enable more accurate Short-Term Load Forecasting (STLF) by identifying intricate temporal correlations, seasonal variations and the stochastic fluctuations in residential demand^39^. They facilitate advanced energy management strategies such as demand response optimization, local storage scheduling and renewable generation balancing in microgrids. However, with these advantages aside, the majority of ML-based prediction models are plagued by their computational complexity, non-scalability and vulnerability to cyber-attacks where data can be tampered^40^. Microgrid environments, where there are large smart meters exchanging real-time telemetry data on communication networks, can greatly detract from prediction accuracy and operational security with such weaknesses. Therefore, the demand is imperative for light construction, cyber-resilient learning frameworks possessing high prediction accuracy with resistance to data tampering or deceptive data injection.

Mathematically, the inconsistencies in the smart meter LF data due to the cyber-attacks are numerically modeled using Benford’s law for significant analysis and accuracy. It asserts that the significance of data is logarithmically distributed rather than equally distributed. With a probability of roughly$\:\:0.3$, the first non-zero number is$\:\:1$. The known Pearson test is a popular method for evaluating Benford^41^. Benford Law improves the accuracy of data in smart meters and specifies the kinds of numbers that should be the first digit in a set of data. The data have been modified if the actual results contradict what Benford Law predicts. Furthermore, by using Benford Law, security increased, and it verifies that smart meter readings are correct. It is a concept for anomaly detection. It can determine that the most commonly starting digit is$\:\:1$, with a decreasing frequency up to$\:\:9$. A technique is used to ensure that the measured frequencies keep pace with theoretical Benford distribution. Grid observations obtained from smart meters are utilized to evaluate stochastic grid computations using a cyber-attack model^42^. The distance among measurements and the confidence ellipse placed around the expected value is a more effective way to identify anomalous data in the power grid. The approach is evaluated by comparing it to the efficient test for identifying faulty data and the chi-square test^43^. Smart metering is a crucial technological element of the smart grid; the privacy of the electrical consumption data must be preserved.

For increasing the capability and scalability of LF approach with residential microgrids, the emergence of deep recurrent mixer models provides significantly advanced LF profile in the distribution networks by combining the temporal learning capabilities of recurrent architectures with the feature-mixing strengths of transformer-based layers^44^. These models, commonly built on foundations of RNNs, LSTM units and Temporal Mixer Block (TMB) is capable of retaining long-temporal dependencies and spatial correlations between dispersed nodes in the grid at the same time. Temporal recurrence combined with channel-wise and token-mixing mechanisms in these models guarantee improved accuracy and the generalization for ALLF and ILF approaches^45,46^. Their application to distribution-level data enables more reliable short-term and medium-term predictions, particularly in environments characterized by nonlinear consumption behaviors and fluctuating renewable energy contributions. These developments demonstrate the growing importance of hybrid DL architectures in modern power system analytics^47,48^, inspiring the need for efficient, cyber-resilient forecasting frameworks such as the one proposed in this study.

Reviewing the major contributions in the domain of enhancing the STLF accuracy as the most appropriate approach to deal effectively with the smart meter data utilizing the different modeling schemes, Table 1 highlights vital samples of these accuracy improvement contributions for comprehensive state of art.

**Table 1:** Practical implementations of the impact of SCM on the PV forecasting strategy profile.

Contribution	Strategy framework	References
Integration of STLF and ALLF modeling scheme		
Using an ANN model to forecast the aggregate daily electricity usage of households	Households were initially classified into distinct groups based on their energy consumption patterns ↓ ANN is used to forecast energy usage of each distinct group ↓ Cluster-level forecasts were then effectively aggregated to generate an overall load prediction for entire system	^49^
Using a cluster approach to improve the accuracy of STLF	Households are clustered using a cluster-based ALLF ↓ Electricity consumption of each cluster was individually predicted, and the forecasts were then aggregated	^50^
Developing an innovative ensemble forecasting model that effectively predicts aggregated load profiles	The model initially employs a clustering technique to group profiles with similar characteristics ↓ The individual electricity consumption predictions are made for each cluster using separate forecasting models ↓ The aggregated load is determined by aggregating the predicted cluster-level consumption values	^51^
Conducting STLF at the aggregated level using a variety of methods	Starting with training data using the analysis of LRM ↓ After extracting the results, the data will be trained again with Deep Learning Analysis (DLA) for accurate operation	^52^
Proposing a novel model to improve the forecasting accuracy of electricity consumption at the aggregated level	Grouping similar time series to identify the underlying patterns in the data and making more accurate predictions ↓ Using the representation techniques allowed for the efficient processing of a large dataset	^34^
Employing a SVR model for enhancing the profile of STLF	Training the extracted data from a cumulative load of about $\:3639$ smart meters to take a prediction action using the analysis profile of SVR model	^29^
Investigating the effectiveness of two unsupervised ensemble learning algorithms in evaluating the performance at the aggregated or clustered level	Employing the clustering approach based on the bootstrap aggregation learning algorithm ↓ Then, the applied studies are conducted using density-based clustering learning algorithm ↓ The aggregated load is determined by comparing the extracted data from the two integrated models	^53^
Integration of STLF and ILF modeling scheme		
Taking advantage of DLA capabilities at the individual and aggregated levels	A deep RNN model was developed and evaluated on a dataset of $\:100$ households ↓ At an aggregated level, real data from New England is used and trained for effectiveness assessment	^54^
Proposing method leverages K-Mean clustering to group residential customers with comparable load consumption patterns into distinct clusters	This clustering step enables the identification of representative load profiles for each cluster which are then used to train a DLA model for LF ↓ Experimental results obtained from evaluating this methodology on residential load data demonstrate its superior performance compared to existing benchmark methods	^55^
Presenting an ensemble electricity consumption forecasting model at different scales	The model’s architecture integrates two advanced DLA techniques; LSTM unit and GRU to effectively capture intricate temporal patterns in the electricity load profile ↓ The model employs the K-MEANS clustering algorithm to group historical electricity consumption data at the house level based on electricity profiles ↓ These clusters are then aggregated according to the consumption profiles of consumers utilizing these data at the building and floor level to train the ensemble models	^56^

Deeping on the significant impact of ILF accuracy considering the manuscript scope, smart meters use communication networks to send the consumers’ load measurement data remotely to electricity suppliers in order to accurately forecast future demand, making LF system categorically vulnerable to cyber-attacks. Consumers’ telemetry data sent from smart meters are vulnerable to cyber-attacks due to vulnerabilities in communication system media such as the fiber-optic cables, microwaves, power-line carriers and radio communication^57^. Cyber-assailants can hack into communication system media and then tamper with consumers’ telemetry measurements that are sent from smart meters to system operators as in the case of cyber-attacks on the Ukrainian power network in $\:2015$ and cyber-attacks on the power supply system of the Indian city of Mumbai in $\:2020$^57^. As a result, it is no longer suitable to suppose that consumers’ telemetry data obtained by smart meters are always real data.

Considering the Indian residential community, The P2P is utilized to face accurately the cyber-attacks at lower operating expenses. It is challenging to characterize the transactions at the physical network levels when P2P electricity trading is utilized in electrical networks^58^. The Internet of Things (IOT) is gaining popularity and innovative micro-level P2P energy trading is growing as the electrical grid quickly shifts to a smart grid paradigm. Globally, about $\:\text{28,000}\:TWh$ of electricity are required each year. It is anticipated that this energy requirement can be doubled. The excess demand for electricity varies by region and country. It is reliant on both growing populations and electricity production. It is anticipated that the value will rise to$\:\:330\:GW$. A secure peer-to-peer energy trading system consists of two layers and blockchain foundation. Verification and safe energy trading comprise the two layers. A mutual authentication process is employed in the authentication layer to protect the suggested model against impersonation attacks^59^. In addition to reducing risks and a significant amount of labor with material resources, blockchain improves transparency of energy transactions. Energy trading can be done with traditional P2P trading systems, but there are drawbacks, such as energy losses that happen during transmission and distribution, which reduce efficiency.

Due to the hazardous security vulnerability in smart meter LF operation, the mathematical modeling and controlling scenarios for these meters become more complicated with several measurement constraints to be handled. Consequently, there is an imperative necessity for the application of sophisticated methods and instruments to solve these multiple complex mathematical models. In this manuscript, the proposed sophisticated methodology depends generally on predicting the individual electricity usage based on the smart metering profile in a manageable computational time. Specifically, this sophisticated methodology comprises a novel pragmatic approach based on integrating a clustering technique with Neural Network (NN) method to obtain the prediction values for each meter.

The utilization of clustering techniques in ILF studies has gained significant popularity in the recent decades due to its smart capability to reduce the amount of data required to train a given machine learning model. The clustering concept is not a spur of the moment or a new scientific term, the previous studies focused mainly on its significant impact on enhancing the ILF performance. One of the common attempts was the development of a forecasting approach for electricity load of university buildings by integrating ARIMA model with clustering strategy^27^. This attempt employed a K-MEANS clustering concept to group data from an entire year including the forecasting day into distinct clusters. Subsequently, the ARIMA model was utilized to predict the cluster-level electricity consumption peaks. Another attempt of significant reputation was development of various electric load clustering methods to uncover the underlying structure of load data, identify distinct load patterns and categorize constituents of the overall load^35^. The other contributions focused on the utilization of several well-known clustering algorithms on embedded time series data^60^. The contributions demonstrated superior performance of these well-established clustering algorithms compared to traditional methods. Previous contributions highlights the main challenges for firming the relation between ILF studies and clustering algorithms in the higher computational time. Almost all the attempts depended on the aggregation of load data to overcome the significant increase in the processing time of integrated model of ILF with clustering algorithms^61^. However, this approach fails to provide granular insights into individual-level electricity consumption patterns.

**Fig. 1:**
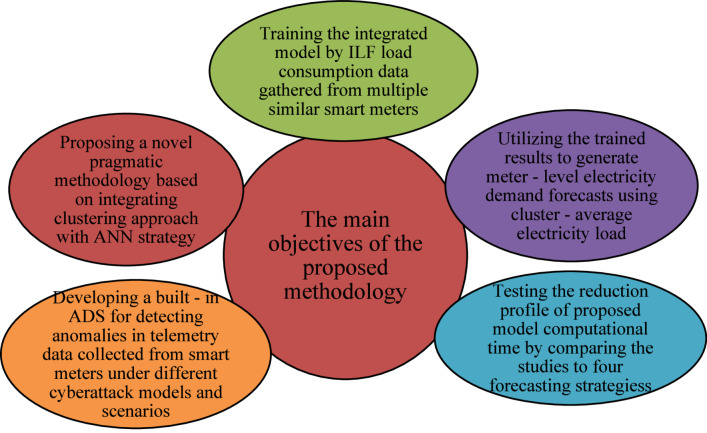
The bullet functions of the manuscript proposed methodology.

To address the double challenge of per-client smart meter prediction in terms of computational cost and susceptibility to cyber-attacks, this paper introduces a combined and sophisticated clustering, neural network-based forecasting, and an anomaly detection approach. The process begins with the clustering of those smart meters with common consumption behaviors by the K-MEANS algorithm to support processing scalability and model redundancy elimination. Cluster-level average usage behaviors are forecasted and utilized for the initialization of NN models, which are later optimized using real per-client data to enhance the accuracy of the forecasts. A PCA–OCSVM based anomaly detecting module is also integrated in the process, which prevents falsified data, to make the system robust against false data injection attacks in telemetered data. The process is comprehensively tested using a real data set of five months of per-hour electricity consumption of $\:\text{2,089}$ clients using smart metering technology. This real data was obtained from the Electrical Distribution Sector (EDS) of Suez Canal Authority (SCA) in Egypt representing a vital source for enriching the scientific studies^62^. This dataset contains the anonymized hourly electricity consumption collected within the period starts from $\:July\:2023$ to $\:December$ $\:2023$. The entire workflow and functional objectives are graphically presented in Fig. 1, summarizing key design visions of the developed methodology. The main contributions of this work are as follows:

1. Development of a scalable KMEANS–NN forecasting framework that groups the meters with similar usage patterns and constructs aggregate forecasting models, cutting down computational overhead drastically without loss of resolution of forecasts at unit-level.
2. Design of a dual-input neural network strategy, where the model is trained on both the cluster mean values and the raw individual meter readings, so the network can learn common usage behavior as well as fine-grained variations to enable more accurate LF.
3. Integration of a robust Anomaly Detection Scheme (ADS) that applies PCA and OCSVM, effectively detecting anomalous data before its interference with the processes of prediction.
4. Outstanding detection performance under the presence of cyber-attack scenarios, where the ADS achieved $\:99.3\%$ overall accuracy, $\:100\%$ sensitivity, $\:98.62\%$ precision, and detected $\:98.6\%$ of compromised telemetry data for scaling, ramping, and random attack types.
5. Improved accuracy and speed of prediction, with the proposed framework providing up to $\:25.6\%\:$decrease in Mean Arctangent Absolute Percentage Error (MAAPE) and cutting down run time from days to minutes, which is apt for real-time operation in the smart grid.
6. Extensive real-world validation using a full $\:\text{2,089}$-unit dataset over five months and robust validation of different forecasting models including ARIMA, Conditional Inference Trees (CTREE), Multi-Layer Perceptron (MLP) and Neural Network Autoregressive (NNETAR).

**Fig. 2:**
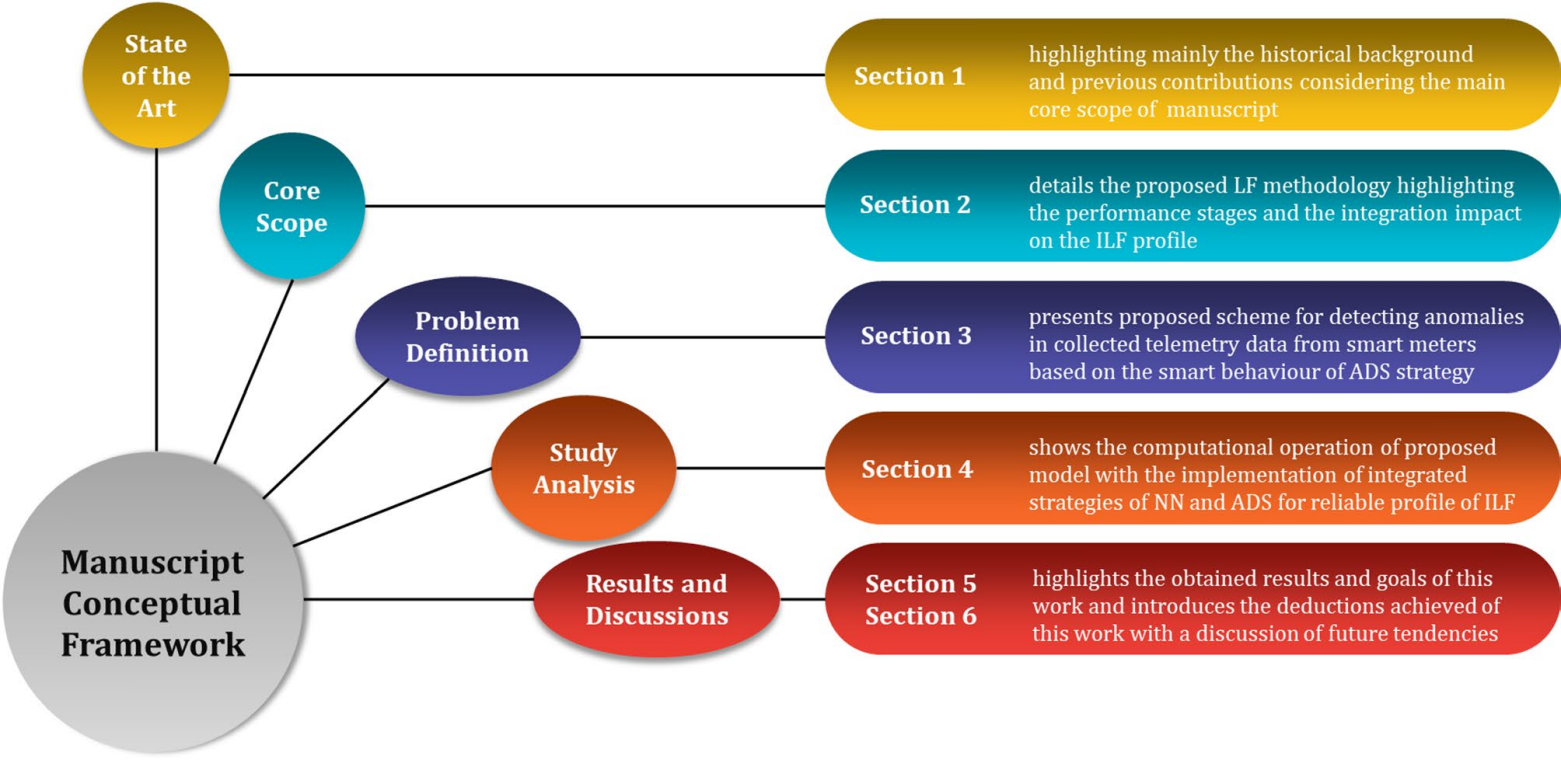
Manuscript conceptual framework.

Reaching the paper framework destination, the structure of this paper is organized in a conceptual manner starting from the state of art stage until reaching the highlighted scores. In this regard, the mind map shown in Fig. 2 traces the work framework of this manuscript.

## Proposed pragmatic methodology for enhancing ILF profile with smart meter data

**Fig. 3:**
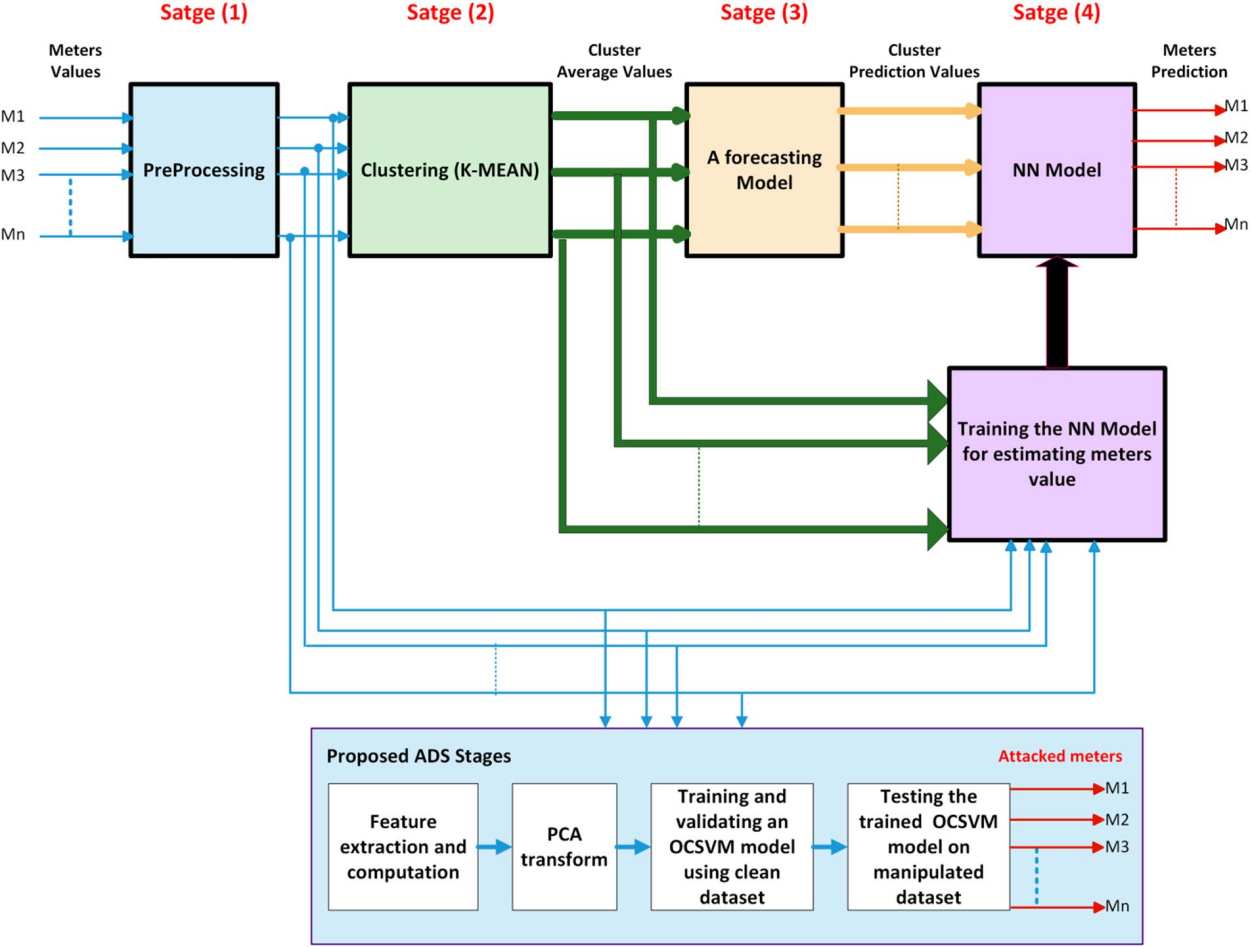
Block representation of the proposed methodology.

Significantly, the proposed methodology in this manuscript is designed to forecast the electricity demand for individual smart meters separately rather than relying on the aggregated predictions while maintaining an acceptable computational efficiency. To reduce the computational time, a forecasting model for each cluster is built to mimic the forecasting model for each meter. This imitating process passes through four stages. Firstly, the meters with similar behavior are grouped using the K-MEANS technique. For each cluster, the average calculations are performed for the operating meters that belong to this cluster. Once the clusters are formed, this methodology constructs a forecasting model for each cluster. These models are trained to predict the cluster-average electricity load for their respective clusters. In the final stage, the proposed NN model is employed to obtain individual-level electricity consumption predictions for each meter. NN model utilizes the prediction values generated by the cluster-specific forecasting models and the characteristics of each meter load profile to generate precise individual-level forecasts. These explained stages are block diagramed with bullet expressions and data orientation as shown in Fig. 2. This figure highlights the four stages of the proposed methodology as follows: data preprocessing; clustering similar meters; forecasting the cluster values and individual-level prediction. Additionally, the proposed ADS according to Fig. 3 consists of four stages as follows: feature extraction and computation stage for each cluster dataset, dimensionality reduction of features using the PCA transformation, training and validating of OCSVM model using a clean dataset and finally testing the profile of the trained OCSVM model on a contaminated$\:/$manipulated dataset resulting from different cyber-attack scenarios.

As the main core of this manuscript, the highlighted block diagram stages in Fig. 2 are deeply discussed in order to provide a comprehensive visualization for the operational behaviour of this methodology. This deep analysis focuses on the theoretical concepts of each stage reviewing the main strategic frameworks of these stages for processing their desired functions.

### Stage 1: data preparation and preprocessing

**Fig. 4:**
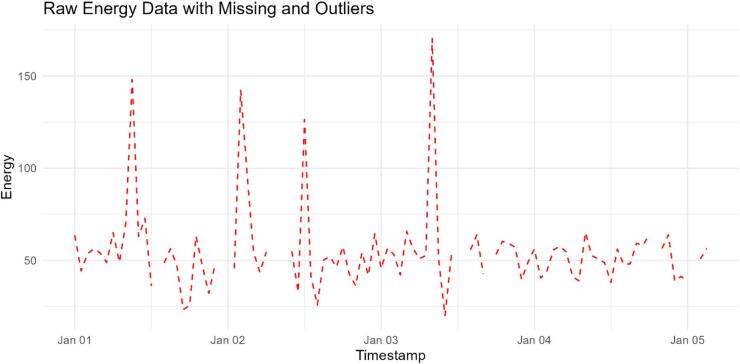
Visualization of input smart meter data before preprocessing highlighting the missing values and outliers.

**Fig. 5:**
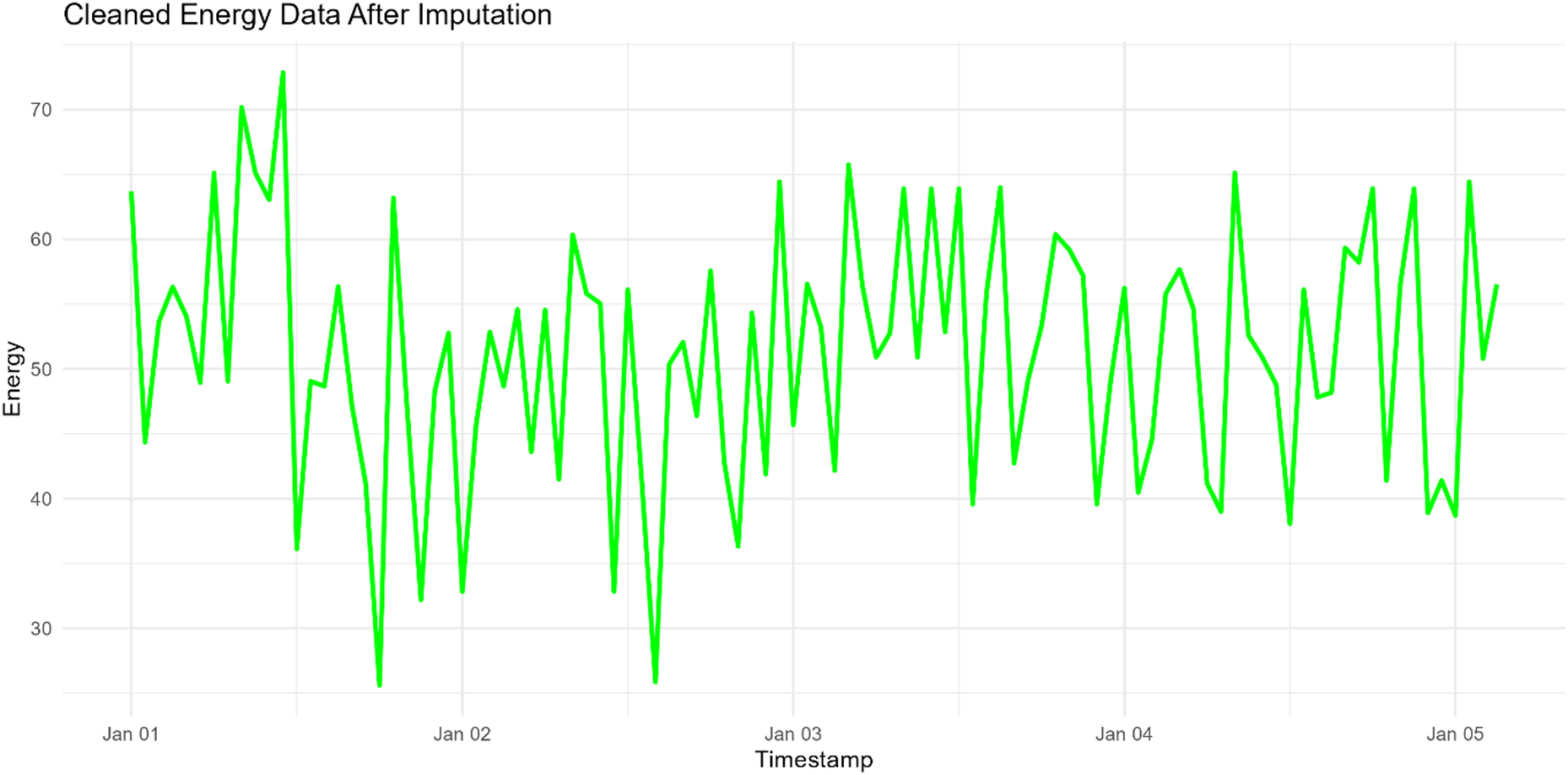
Visualization of cleaned smart meter data after applying outlier detection and imputation.

This stage involves the loading, preprocessing and splitting approaches of the smart meters’ energy data. Data preparation is a crucial step in any analytical endeavor due to the challenges of analyzing raw data. This stage is concerned with the data recognition detecting the missing ones. In this regard, outliers that refer to data points deviate significantly from the rest of the dataset are one of the issues that must be addressed firstly in this stage^31,63^. Outliers that are defined as missing data should be accurately identified and then replaced with appropriate substitute values. This stage employs three outlier detection methods: box plot, statistical strategies and novel specialized univariate outlier detection methods^63–65^. This research uses the impact of Multivariate Imputation Chained Equations (MICE) algorithm for outlier replacement^66,67^. In this regard, the smart meters’ energy data is split into the training and testing sets to train and evaluate the performance parameters of the proposed method. For strengthen this stage, the visualization plot Fig. 4 illustrates the original smart meter energy readings where the missing values and extreme outliers are clearly visible. Furthermore, after applying pre-processing, the visual diagram Fig. 5 shows the cleaned dataset with smooth and consistent energy readings.

### Stage 2: meter clustering

In this stage, similar smart meters are grouped into a number of clusters using the K-MEANS algorithm. The K-MEANS approach is a widely used unsupervised machine learning algorithm that partitions a given dataset into a predefined number of clusters^68^. The K-MEANS algorithm groups the data based on their proximity to predefined cluster centers. Each cluster center represents the average of the data points that are currently assigned to that cluster. Then, K-MEANS algorithm iterates for a countered loop until the meters are no longer being reassigned to different clusters. The operation of the K-MEANS algorithm in this stage is subjected to the consumption pattern profiles of these gathered smart meters to enhance the precision of LF models. These consumption patterns of smart meters can be characterized by a number of features such as the total and average consumption across different hours and days of the week. The K-MEANS algorithm can be used to identify clusters of smart meters with highly correlated consumption patterns for all of these features. The structural methodology framework of this stage with the consumption pattern profile comprises the following processes:

#### Extracting features for clustering smart meters

The precise categorization of smart meters considering their consumption patterns is crucial for accurate LF profile. To achieve this process, a comprehensive set of distinctive features is employed encompassing various metrics capturing the nuanced energy usage patterns of the smart meters. These characteristics include total energy consumption aggregated from hourly energy values, average energy consumption over diverse timeframes (Hourly; Daily; Weekly; Monthly or Yearly), average consumption distinctions between weekends and business days and consumption averages across different daily time periods. By integrating these multifaceted dimensions into K-MEANS software, the capability to group smart meters according to their consumption patterns is significantly enhanced facilitating definitely more precise and streamlined LF profile. This process is considered as the mathematical modeling stage of the clustering algorithm. In this regard, these extracted features are comprehensively represented based on the following mathematical formulas for each estimated step:

##### Total energy consumption

The total energy consumption for each smart meter is mainly calculated by summing up the hourly energy consumption values. This feature that represents the overall energy usage of smart meter is expressed as:

1 $$\:{TKWh}_{i}=\sum\:_{j=1}^{H}{x}_{ij}\:\:\:\:$$

where, $\:\varvec{H}$ represents the total number of hours and $\:{\varvec{x}}_{\varvec{i}\varvec{j}}$ represents the energy consumption of $\:{\varvec{i}}^{\varvec{t}\varvec{h}}$ smart meters at $\:{\varvec{j}}^{\varvec{t}\varvec{h}}$ specific time step.

##### Average energy consumption

The average energy consumption is calculated by dividing the obtained total energy consumption within the corresponding defined time period (Hourly; Daily; Weekly; Monthly or Yearly). This feature provides insights into the average energy usage patterns of the smart meters over different time scales. The total number of hours$\:\:\left(\varvec{H}\right)$, days$\:\:\left(\varvec{D}\right)$, weeks$\:\:\left(\varvec{W}\right)$, months$\:\:\left(\varvec{M}\right)$ and years $\:\left(\varvec{Y}\right)$ are used as the denominators in the respective average energy consumption calculations as expressed in the following formulas:

2 $$\:{avergehourly:\:\:\:Avg}_{i}=\:\frac{{TKWh}_{i}}{H}\:\:\:\:\:\:\:\:\:\:$$

3 $$\:{avergedaily:\:\:\:AvgD}_{i}=\:\frac{{TKWh}_{i}}{D}\:$$

4 $$\:{avergeweekly:\:\:\:AvgW}_{i}=\:\frac{{TKWh}_{i}}{W}\:\:\:\:\:\:\:\:\:$$

5 $$\:{avergemonthly:\:\:\:AvgM}_{i}=\:\frac{{TKWh}_{i}}{M}\:\:\:\:\:$$

6 $$\:{avergeyearly:\:\:\:AvgY}_{i}=\:\frac{{TKWh}_{i}}{Y}\:\:\:\:\:\:\:\:\:\:\:\:\:\:\:$$

##### Average weekly day consumption ($\:\varvec{A}\varvec{v}\varvec{g}\varvec{W}\varvec{D}\varvec{a}\varvec{y})$

Comprehensively, the average energy consumption for each weekday (Saturday to Friday) is calculated by averaging the hourly energy consumption values for that specific weekday. This feature identifies the profile of patterns in the energy usage across weekdays according to the following formula:

7 $$\:{AvgWDay}_{i,k}=\frac{1}{w}\sum\:_{j=1}^{n}{\left.{x}_{ij\:}\right|}_{day=k}\:\:\:\:\:\:\:\:\:\:\:\:\:\:\:\:\:\:$$

where, $\:\varvec{k}$ is the weekdays operator ranged within the interval $\:[1:7]$ considering the targeted weekdays.

Clearly, when the operator$\:\:\varvec{k}=\:1$, the day is chosen to be ***“Saturday”*** and the energy data is aggregated every Saturday. In sequential step, this aggregation is divided by number of weeks.

##### Average weekend and business day consumption

The average energy consumption for weekend (Saturday and Sunday) and workdays (Monday to Friday) are calculated by averaging the hourly energy consumption values for respective time periods. This vital feature distinguishes among energy usage patterns on weekends and workdays as follows:

8 $$\:{weekEnd}_{i}=\frac{1}{2}\sum\:_{k=1}^{2}\:{\left.{AvgWDay}_{i,k}\right|}_{day=k}\:\:\:\:\:\:\:\:\:\:\:$$

9 $$\:{WorkDay}_{i}=\frac{1}{5}\sum\:_{k=3}^{7}{\left.{\:AvgWDay}_{i,k}\right|}_{day=k}\:\:\:$$

##### Average across daily time periods consumption

- To analyze the energy consumption patterns across different time periods of the day, the average energy consumption is calculated for five distinct time intervals:
- Night Period$\:\:[10\:P.M.\:to\:6\:A.\:M.]$: represents typically low-energy consumption phase of the day when most household activities are minimal.
- Late Afternoon Period$\:\:[6\:P.M.\:to\:9\:P.\:M.]$: corresponds to the period when cooking and evening activities typically increase energy demand.
- Early Afternoon Period$\:\:[2\:P.M.\:to\:5\:P.\:M.]$: coincides with the time when household activities such as cooking, laundry and recreational activities are prevalent.
- Morning Period$\:\:[10\:A.M.\:to\:1\:P.\:M.]$: represents the moderate energy consumption period that is typically associated with activities like breakfast preparation and household chores.
- Early Morning Period$\:\:[7\:A.M.\:to\:9\:A.\:M.]$: coincides with the time when energy consumption increases due to activities like showering, preparing for work or school, and using appliances.

The average energy consumption for each of these time periods can be calculated as follows:

- *Total Load Daily*:
  10 $$\:{Tothour}_{i,\:h}=\sum\:_{j=h}^{n}{x}_{ij\:}\:\:\:\:\:j=h,\:h+24,\dots\:.,n$$
  where, $\:h$ is the hourly day. When$\:\:h="1A.M."$, energy data is aggregated every hour at $\:"1A.M."$ of day. After that, this aggregation is divided by the number of hours based on daily periods.
- *Average Load at Early Morning (*$\:Avg\_EM)$ $\:[7\:to\:9\:A.M.]$:
  11 $$\:{Avg\_EM}_{i}=\frac{1}{3}\sum\:_{h=7}^{9}{Tothour}_{i,\:h}$$
- *Average Load at Morning (*$\:Avg\_M)$ $\:[10\:A.M.\:to\:1\:P.M.]$:
  12 $$\:{Avg\_M}_{i}=\frac{1}{4}\sum\:_{h=10}^{13}{Tothour}_{i,\:h}$$
- *Average Load at Early Afternoon (*$\:Avg\_EAN$*)* $\:[2\:to\:5\:P.M.]$:
  13 $$\:{Avg\_EAN}_{i}=\frac{1}{4}\sum\:_{h=14}^{17}{Tothour}_{i,\:h}$$
- *Average Load at Late Afternoon (*$\:Avg\_LAN)$ $\:[6\:to\:9\:P.M.]$:
  14 $$\:{Avg\_LAN}_{i}=\frac{1}{4}\sum\:_{h=18}^{21}{Tothour}_{i,\:h}$$
- *Average Load at Night (*$\:Avg\_N$*)* $\:[10\:P.M.\:to\:6\:A.M.]$,
  15 $$\:{Avg\_N}_{i}=\frac{1}{3}\sum\:_{h=22}^{24}{Tothour}_{i,\:h}+\frac{1}{6}\sum\:_{h=1}^{6}{Tothour}_{i,\:h}$$

#### Normalization

In order to ensure effective clustering profile using the K-MEANS algorithm that is sensitive to the distances between the data points, the extracted features should be normalized to fall within a comparable range. This step is achieved by employing the min-max normalization function that scales down each feature to a range between $\:0$ and $\:1$ as follows:

16 $$\:Xnormalized\:=\frac{{x}_{i}-min\left(x\right)}{max\left(x\right)-min\left(x\right)}\:\:\:\:\:\:\:\:\:\:$$

where, $\:{\varvec{x}}_{\varvec{i}}$ is an observation operator and $\:\varvec{x}$ is the sample data.

#### Determining the optimal number of clusters

Determining the appropriate number of clusters is a crucial issue in K-MEANS clustering. Gap Statistic Method (GSM) is one of the most popular methods for selecting the optimum number of clusters^69^. The GSM is a statistical technique employed to determine the optimal number of clusters in a dataset. It compares the Within-Cluster Sum of Squares (WCSS) of the actual data to the WCSS of a referenced distribution generated under a null hypothesis of no clusters. The optimal number of clusters is identified as the value that maximizes the gap statistic indicating that the WCSS of the actual data is significantly lower than that of the reference distribution suggesting a meaningful clustering structure.

### Stage 3: cluster-level forecasting

In the preceding stage, the unsupervised K-MEANS algorithm is utilized to group the meters into a discrete number of clusters considering their consumption load patterns. This process involves iteratively assigning meters to the nearest cluster centroid aiming to minimize the within-cluster variance. Once the clustering approach is completed, each cluster represents a distinct group of meters with similar electricity consumption patterns. In the preparation for LF, the mean electricity consumption values are determined for each identified cluster. These average values represent collective electricity consumption behavior of the meters within that cluster. Utilizing these average values, the forecasting process can be simplified, and computational efficiency can be improved.

The use of average cluster values instead of individual meter values is particularly beneficial in the large datasets where forecasting individual meters can be computationally expensive. Focusing on the average consumption of each cluster, the forecasting model can capture the overall consumption trends of similar meters without the need to model each meter individually. This approach not only reduces computational burden but also improves the generalizability of forecasting models. In this study, four forecasting methods including: ARIMA, CTREE, MLP and NNETAR are utilized to forecast the future values for each cluster separately^70–73^. Overall, the clustering and averaging process play vital roles in preparing data for accurate and efficient ILF profile. By grouping similar meters and using average cluster values, ILF can focus on the underlying consumption patterns of different meter types for robust and transferable forecasting results.

### Stage 4: individual-level prediction

NN is a powerful and versatile machine learning tool that can be utilized in various applications in order to construct a forecasting model^74–76^. The ability to capture complex non-linear relationships within data makes this tool particularly well-suited for modeling intricate dynamic patterns inherent in the time series data. NNs are defined as a network system with *“neurons”* that communicate with each other. The connections have numerical weights that enable neural nets to learn and adapt to inputs. Linear and non-linear relationships can be handled by NNs. As a result, NNs are utilized in many different industries to carry out complex tasks or approaches such as classification, pattern recognition and prediction^77–79^.

**Fig. 6:**
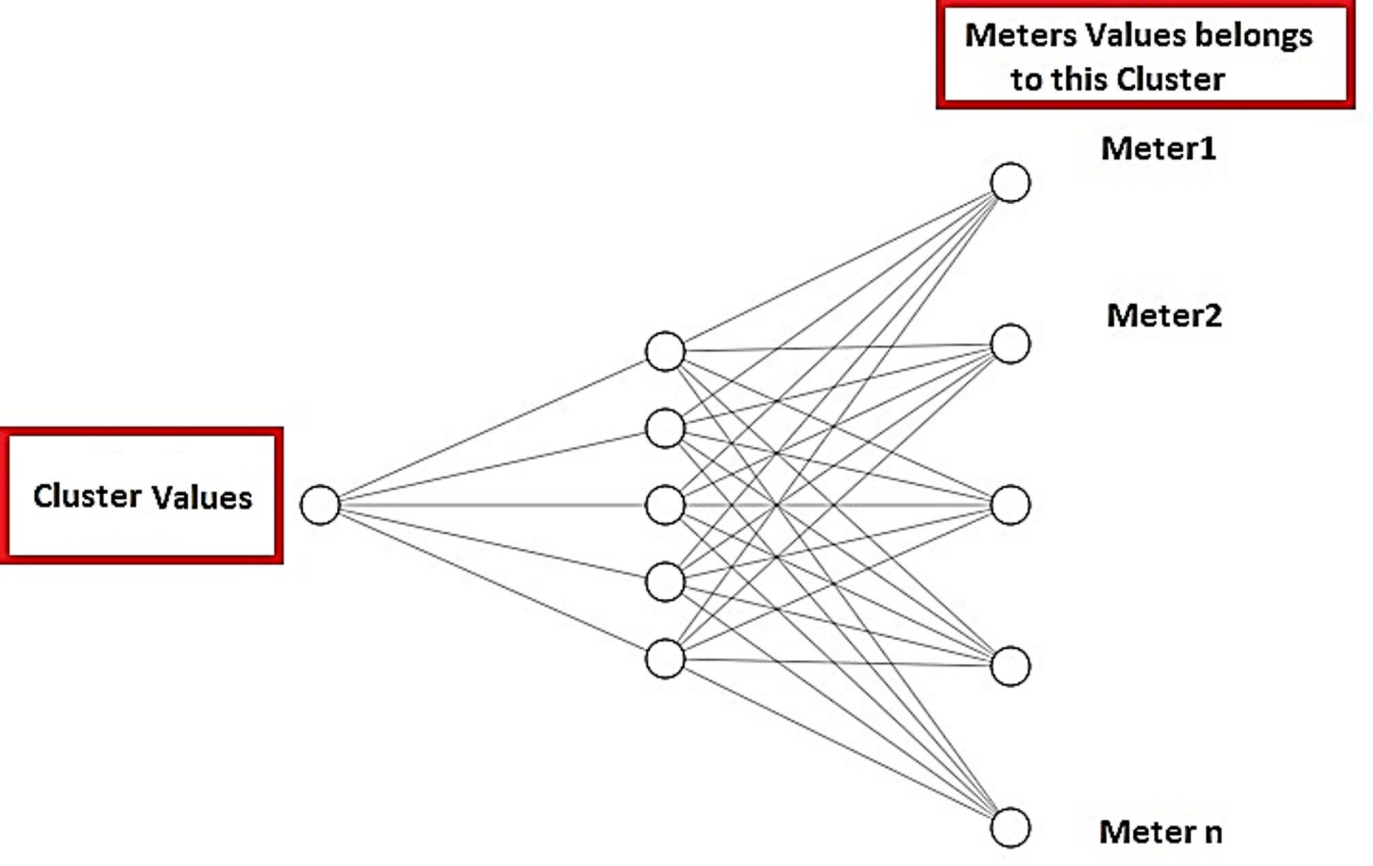
The NN structure for estimating the meter values form its cluster prediction values.

Building a mathematical model that can accurately predict future electricity consumption for each meter is a complex task due to the non-linearity and multifaceted nature of the data. To address this challenge, NN model is constructed in this phase to estimate the future electricity demand at an individual meter level. Instead of building a separate NN model for each meter, a single NN model per cluster is constructed to estimate the values of the meters within that cluster as shown in Fig. 6. This approach smartly reduces the total number of trained models, thereby reducing the computational time for training and inference. The proposed NN model is designed to take prediction values of each cluster from the previous stage and estimate the meters’ values. The proposed NN model consists of three layers (input, hidden and output) as defined in Fig. 3. The input layer presents the prediction values for each cluster. The hidden layer with five sigmoid neurons is used to capture the non-linear relationship between the input and output layers. The output layer contains the number of meters that belong to that cluster. The number of output neurons in each NN model corresponds to the number of meters within that cluster. The average electricity consumption of the cluster and actual values of individual meters within that cluster are employed as inputs for training the respective NN models. Regarding training stage, NN model is utilized to generate prediction values for each meter based on cluster-level prediction values obtained from forecasting model in stage$\:\:3$.

## Cyber-attacks against smart meter telemetry data

More sophisticated and skilled hackers may compromise a system, covertly alter the vital data with only minor modifications and introduce significant inaccuracies into the forecasting output^80^. As the LF techniques rely significantly on the historical load data to build, train and update or retrain forecasting models, they may be easily vulnerable to cyber-attacks targeting the consumers’ telemetry data collected from distributed meters^81^. In this regard, the proposed methodology in this paper depends on the defensive impact of ADS strategy for detecting the anomalous/false data injected into telemetry data collected from smart meters.‎ Firstly, cyber-attack models/scenarios against telemetry data of smart meters are presented. Secondly, the features used in the proposed ADS model are defined. Finally, the proposed ADS model based on a combination of PCA and OCSVM is described.

### Cyber-attack models against smart meter telemetry data

One of the main goals of the cyber-attacks against LF systems is to manipulate smart meter telemetry data making the electric load power profile differ significantly in the training data stage from its actual profile fed into the LF model. As a result, bad decisions could be made based on the performed forecasts. Scaling, ramping and random attacks are three most common types of cyber-attacks against LF profile^81,82^.

#### Scaling attacks

In this type, smart meter telemetry data are multiplied by a low$\:/$high scaling attack coefficient ($\:1+{\varvec{k}}_{\varvec{s}}$) during specific time intervals. The scaling attack model can be formulated as follows:

17 $$\:{x}_{i,t}^{a}=\left(1+{k}_{s}\right){x}_{i,t},\:\:\:\:\:\:\:\:\:{for\:\:t}_{i}<t<{t}_{e}\:\:\:\:\:$$

where, $\:{\varvec{x}}_{\varvec{i},\varvec{t}}^{\varvec{a}}$ is the attacked$\:/$manipulated telemetry data representing load power of $\:{\varvec{i}}^{\varvec{t}\varvec{h}}$ smart meter during specific time intervals, $\:{\varvec{x}}_{\varvec{i},\varvec{t}}$ represents the original$\:/$clean telemetry data ‎of $\:{\varvec{i}}^{\varvec{t}\varvec{h}}$ smart meter, $\:{\varvec{k}}_{\varvec{s}}$ is scaling attack operator that ranged within interval **(**$\:{-1<\varvec{k}}_{\varvec{s}}<\varvec{\infty\:}$**)**, $\:{\varvec{t}}_{\varvec{i}}$ and $\:{\varvec{t}}_{\varvec{e}}$ are the limits of scaling attack time.

#### Ramping attacks

Ramping attack occurs in two intervals. In the first interval, the telemetry data gradually increased until reaching a specific peak value. In the second interval, this data is gradually minimized from the reached peak value. The following relationships represent the ramping attack mathematical modeling:

18 $$\:{x}_{i,t}^{a}=\left\{\begin{array}{c}\left[1+{k}_{r}\left(t-{t}_{i}\right)\right]{x}_{i,t},\:\:{\:\:\:\:\:t}_{i}<t\le\:\frac{{{t}_{i}+t}_{e}}{2}\:\\\:\left[1+{k}_{r}\left({t}_{e}-t\right)\right]{x}_{i,t},\:\:\:\:\:\:\frac{{{t}_{i}+t}_{e}}{2}\le\:t<{t}_{e}\end{array}\right.\:\:$$

where, $\:{\varvec{k}}_{\varvec{r}}$ refers to the ramping attack coefficient.

#### Random attacks

In this type, a random positive value **(**$\:{\varvec{k}}_{\varvec{d}}{\varvec{R}}_{\varvec{t}}$**)** is injected$\:/$added to telemetry data of $\:{\varvec{i}}^{\varvec{t}\varvec{h}}\:$smart meter during specific time periods starting from $\:{\varvec{t}}_{\varvec{i}}$ to$\:\:{\varvec{t}}_{\varvec{e}}$. The random attack model can be formulated as follows:

19 $$\:{x}_{i,t}^{a}={x}_{i,t}+{k}_{d}{R}_{t},\:\:\:\:\:\:\:\:\:{for\:\:t}_{i}<t<{t}_{e}\:\:\:\:$$

where, $\:{\varvec{k}}_{\varvec{d}}$ is the scale ‎parameter of the random attack and $\:{\varvec{R}}_{\varvec{t}}$ is a positive random number generator.

### Proposed ADS for detecting anomalous data

Currently, several anomaly detection algorithms in telemetry are utilized^82^. Telemetry data anomaly detection has gained a lot of attention with the rapid development of Artificial Intelligence (AI) strategies, particularly the ML techniques^81,83^. The semi-supervised or unsupervised ML methods such as isolation forest algorithm, ‎OCSVM and local outlier factor are emphasized to be a better option to address the problem of data requirements as supervised methods need two-class labeled data for model training that is not available in some clean telemetry data. In addition, clean telemetry data of smart meters can be easily available instead of generating a dataset containing samples of cyber-attacks that may be impractical and ineffective in some operational cases. In this regard, OCSVM that is defined as an unsupervised anomaly detection method, has been extensively employed in numerous applications due to its resilience, robustness, high speed of training as well as testing and exceptional accuracy with a minimal number of samples^83,84^. Due to unique features, OCSVM model can be trained on uncontaminated or clean data without including anomalous data. Proposed ADS is based on the integration of PCA with OCSVM model‎.

The smart meters are clustered according to similar profiles of their consumption. So, for each cluster, an ADS model is constructed and trained that can then detect anomalies in the telemetry data of each smart meter included in that cluster. This action reduces significantly the computational burden and time.

#### Features used in the proposed ADS

Accurate detection of anomalies in smart meter telemetry data depends on their consumption patterns. To achieve this step, a set of features is selected to recognize the energy usage patterns of smart meters. The desired goal of selecting features for ADS model is to maximize the correlation of these features with the consumption patterns of clustered smart meters.‎ In contrast of clean$\:/$normal telemetry data, noticeable changes in the features of attacked telemetry data can be observed during cyber-attacks. ‎For each cluster, clean$\:/$normal telemetry dataset of smart meters with similar consumption profiles is utilized to determine seven proposed features. These features include the following mathematical metrics: mean value, standard deviation value, root mean square value, crest factor, impulse factor, kurtosis factor and shape factor for clean telemetry data representing consumption patterns of these smart meters. These features are extracted and computed by using Diagnostic Feature Designer (DFD) application in MATLAB environment^85^. This application enables the developers to trace and extract detailed features graphically to make a precise identification for an event, parameter or issue. Significantly, this stage forms a complete visualization of the security system and corrective actions for cyber-attacks considering the topological profile of trained data in addition to its response capability to the different estimated features.

#### PCA–OCSVM based ADS

The selected feature data is transformed by PCA from the original, large-dimensional space into a new smaller-dimensional space^86^. The transformed feature data has the following vital properties^87^:

- The dimensions of the transformed features are reduced that makes the ADS consume less time in the training and testing process.
- The correlation among the transformed features’ data is reduced, that improves the classification accuracy of the proposed ADS.
- The transformed features are ordered based on their data importance and relevance.

The mathematical modeling of this transformation feature is expressed using the following formula that maps the original feature data into the defined transformed feature matrix:

20 $$\:S=\:\left(X-\mathcal{M}\right)*{\rm\:K}\:\:\:\:\:\:\:\:\:\:$$

where, $\:\mathcal{M}$ and $\:\varvec{K}$ are the computed mean of the original features and PCA coefficients to transform the original features into the lower-dimensional features respectively. $\:\mathcal{M}$ and $\:\varvec{K}$ are determined utilizing the impact of singular value decomposition algorithm^86,88^.

**Fig. 7:**
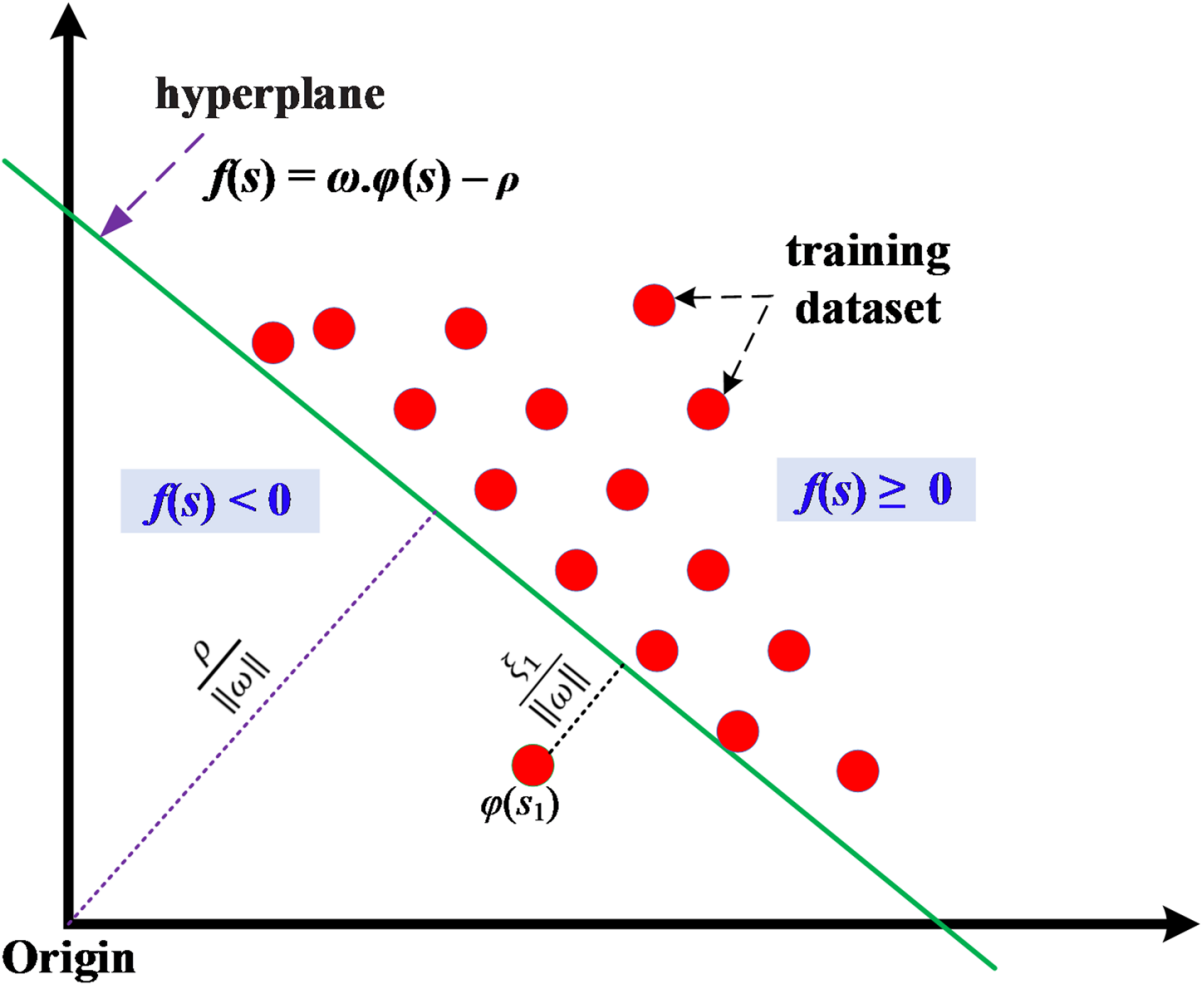
Illustration of OCSVM operational profile.

In this transformation stage, the objective of OCSVM algorithm is to define a hyper-plane isolates the instances of training data representing clean telemetry data of smart meters from the left-over space. As expressed in Fig. 7, OCSVM takes origin as an instance of the second class. OCSVM looks for a hyper-plane that maximizes margin $\:(\varvec{\rho\:}/\varvec{\omega\:})$ between instances of the target class and origin^89,90^. The PCA-transformed feature data $\:\varvec{S}\:=\:[{\varvec{s}}_{1},\:{\varvec{s}}_{2},\:..\:{\varvec{s}}_{\varvec{l}}]$ are mapped into the feature space with higher-dimensions utilizing the transformation objective ‎map $\:\varvec{\phi\:}\left(\varvec{S}\right)$^89,90^. Solving the following optimization problem yields the required hyper-plane equation ($\:\varvec{f}\left(\varvec{s}\right)=\varvec{\omega\:}\bullet\:\varvec{\phi\:}\left(\varvec{s}\right)-\varvec{\rho\:}$) using Lagrange multiplier method^90^.

21 $$\:\underset{\rho\:,\omega\:,\xi\:}{{min}}{\:\:\frac{1}{2}‖\omega\:‖}^{2}\:+\psi\:\sum\:_{i=1}^{l}{\xi\:}_{i}-\rho\:\:\:\:\:\:\:\:\:\:\:\:\:\:\:\:\:$$

22 $$\:\omega\:\bullet\:\phi\:\left({s}_{i}\right)\ge\:\rho\:-{\xi\:}_{i},\:\:{\xi\:}_{i}\ge\:0\:\:\:\:\:\:\:\:\:\:\:\:\:\:\:\:i=1,\:2,\:...,\:l\:\:$$

where, $\:\varvec{\rho\:}$ and $\:\varvec{\omega\:}$ are hyper-plane parameters, slack variable $\:{\varvec{\xi\:}}_{\varvec{i}}$ forces training data instances to be located inside hyper-plane, $\:\varvec{\psi\:}$ operator refers to portion of data samples that is permitted outside targeted class.

If a test data sample $\:\left({\varvec{s}}_{\varvec{t}\varvec{e}\varvec{s}\varvec{t}}\right)$ lies above the hyper-plane level$\:\:(\varvec{\omega\:}\bullet\:\varvec{\phi\:}({\varvec{s}}_{\varvec{t}\varvec{e}\varvec{s}\varvec{t}})\:-\:\varvec{\rho\:}\:\ge\:\:0)$, it is classified as a normal data sample; otherwise, it is classified as an anomalous$\:/$outlier data sample.

Arranging the calculation domain, this paper utilizes a comprehensive dataset of $\:2089$ smart meters. The desired data encompasses $\:18$ months of hourly energy consumption measurements spanning accurately from$\:\:July\:{14}^{th},\:2009\:$to$\:\:December\:{31}^{st},\:2010$ including detailed information on the type of customer (commercial or residential) and the time of day. This actual and practical data is then used to completely implement the proposed pragmatic methodology in order to enhance the ILF profile of smart meters. This implementation and integration profile is discussed in a programmable coding manner.

## Implementation setup

The proposed methodology is implemented using the R-language and conducted on $\:"HP\:xw8600"$ workstation with $\:"Intel\:Xeon"$ and $\:"CPU\:X5450"$ running at $\:3\:GHz$ and $\:2\:GB$ of RAM. R-Language is used for statistical computing and graphics. Two programs are implemented to forecast values of power usage. First program includes proposed KMEANS-NN strategy outlined in Algorithm 1. Subsequently, Algorithm 2 includes pseudo code to utilize NN technique for predicting each meter’s power consumption.

On the other hand, the proposed ADS strategy for detecting anomalies in telemetry data representing the consumer consumption power of smart meters is implemented using the MATLAB environment.

## Results and discussion

### Data description

This study utilizes smart meter data collected under the supervision of EDS of SCA in Egypt. Trials spanned from$\:\:July\:14,\:2022\:$to$\:December\:31,\:2010$ involving over $\:\text{6,000}$ residential and commercial electricity users across Egypt. Dataset includes three key components: half-hourly electricity usage measured in kilowatt-hours$\:\:\left(kWh\right)$, time of consumption and the customer category (residential or business). Notably, the dataset does not contain geographic information about the customers, which limits the integration of weather-related variables. For the purposes of this analysis, only meters with complete data records were considered resulting in a subset of $\:\text{2,089}$ meters. The electricity consumption data for these meters was aggregated to an hourly level to facilitate modeling and analysis.

### General overview and parameterization

The well-trained pragmatic KMEANS-NN methodology is tested to investigate its effect on prediction accuracy and computation time on smart meters. During the test phase, the ILF approach is performed on a set of testing smart meters to assess the methodology’s ability to generate accurate and timely predictions. The performance efficiency of the proposed methodology is assessed utilizing four forecasting methods of ARIMA, CTREE, MLP and NNETAR with the proposed KMEANS-NN strategy and per individual meters (across $\:2089$ smart meters) without applying the KMEANS-NN strategy.

The predictive performance of the proposed model is assessed through a three-step evaluation process:

- Retrieving Prediction Values: Prediction values for each meter are retrieved from model’s output.
- Obtaining Testing Set Values: The actual electricity consumption values for each smart meter from the testing set are obtained.
- Calculating Prediction Accuracy: The prediction accuracy for individual meter is calculated by comparing the prediction outputs of KMEANS-NN with actual electricity consumption values. This accuracy metric quantifies the model’s ability to generate accurate forecasts.

The proposed KMEANS-NN methodology is comprehensively evaluated on a dataset of smart meter energy data. A $\:70\%$ of the time period in the dataset is considered as a training set comprising data from $\:July\:{14}^{th},\:2022\:$to$\:\:July\:{23}^{th},\:2023\:$and a testing set represents sharply the remaining time period of $\:\left(30\%\right)$ encompassing data from $\:July\:{24}^{th},\:2023\:$to$\:\:December\:{31}^{st},\:2023$. To assess the performance of KMEANS-NN methodology in conducting $\:2089$ meters STLF, To comprehensively evaluate the performance of the proposed load forecasting methods, we incorporated six Key Performance Indicators (KPIs): Mean Squared Relative Error (MSRE), Root Mean Squared Relative Error (RMSRE), Mean Absolute Error (MAE), Mean Absolute Relative Error (MARE), Mean Absolute Percentage Error (MAPE), and Root Mean Squared Percentage Error (RMSPE). These metrics were calculated for each forecasting method, both at the individual meter level and using the clustering-based approach. The results in Table 2 demonstrate that the proposed clustering-enhanced models consistently outperform their counterparts across all metrics, indicating improved accuracy and robustness.

(metrics) including MAAPE, Mean Absolute Error (MAE) and Root Mean Square Error (RMSE) are exploited according to the following formulas^31,70,91^:

23 $$\:MAAPE=\frac{1}{n}\sum\:_{t=1}^{n}arctan\left(\left|\frac{{A}_{t-}{F}_{t}}{{A}_{t}}\right|\right)\:\:\:\:\:\:\:\:\:\:\:$$

24 $$\:MAE=\frac{{\sum\:}_{t=1}^{n}|{A}_{t}-{F}_{t}|}{n}\:\:\:\:\:\:\:\:\:\:\:\:$$

25 $$\:RMSE=\sqrt{\frac{{\sum\:}_{t=1}^{n}{({A}_{t}-{F}_{t})}^{2}}{n}}\:\:\:$$

where, $\:{\varvec{A}}_{\varvec{t}}$ represents the actual meter reading value, $\:{\varvec{F}}_{\varvec{t}}$ represents the forecasted load output from the model and $\:\varvec{n}$ denotes the total number of reading samples in the test set.

For the proposed ADS used for detecting cyber-attacks against telemetry data collected from smart meters, the same dataset of smart meter energy data is also used to train and test the ADS model.‎ This collected dataset is randomly split into two folds using the cross-validation method^92^. The first fold is $\:80\%$ of the collected dataset and is used to train the OCSVM model, while the second fold is about $\:20\%$ of the collected dataset and is used to test$\:/$validate the trained OCSVM. Firstly, the selected features of training and testing datasets are extracted and computed using the DFD application within MATLAB domain and then the PCA-transformed features are used to train and validate the OCSVM model.

The profile of the trained OCSVM model is assessed using some indices considering the following metrics:

26 $$\:Accuracy=\:\:\frac{TP+TN}{TP+TN+FN+FP}\:\:\:\:\:\:\:\:$$

27 $$\:Sensitivity\:=\:\:\frac{TP}{TP+FN}\:\:\:\:\:\:\:\:\:\:\:\:\:\:\:\:\:\:\:$$

28 $$\:Precision\:=\:\:TP/(TP+FP)\:\:\:\:\:\:\:\:\:\:\:\:\:\:\:\:\:$$

29 $$\:Specificity\:=\:\:TN/(TN+FP)\:\:\:\:\:\:\:\:\:\:$$

30 $$\:F1-score=\:\:\frac{2\times\:Sensitivity\times\:Precision}{Sensitivity+Precision}\:\:\:$$

where, $\:TP$ and $\:TN$ represent the number of correctly classified normal data and detected anomalous data, respectively. $\:FN$ represents the number of normal data samples that are incorrectly detected as anomalous data and $\:FP$ is the number of anomalous data samples that are incorrectly detected as normal data.

Since the testing dataset is clean$\:/$normal data and not contaminated$\:/$manipulated by cyber-attacks, all test data samples are significantly classified as $\:TP$ or normal samples with the accuracy, sensitivity, precision, specificity and F1-score values of $\:100\%$, $\:100\%$, $\:100\%$, $\:0\%$, and unity, respectively. Then, the trained OCSVM model is effective and reliable for detecting anomalies caused by cyber-attacks. The performance evaluation of proposed ADS under different cyber-attack scenarios will be presented in the next section.

### Findings of the performed studies

This section contains three conducted parts that can be expressed as follows:

- Specifying the optimum cluster number.
- Assessing the operational performance of the proposed four forecasting methods with and without the utilization of KMEANS-NN technique.
- Testing the performance of the proposed ADS under the different types of cyber-attack scenarios.

The effectiveness of proposed KMEANS-NN strategy is thoroughly evaluated using a comprehensive dataset of $\:2089$ smart meters. This extensive evaluation provides robust evidence of the methodology’s generalizability and its ability to accurately predict energy consumption patterns in a large-scale setting.

#### Specifying the optimal cluster number

**Fig. 8:**
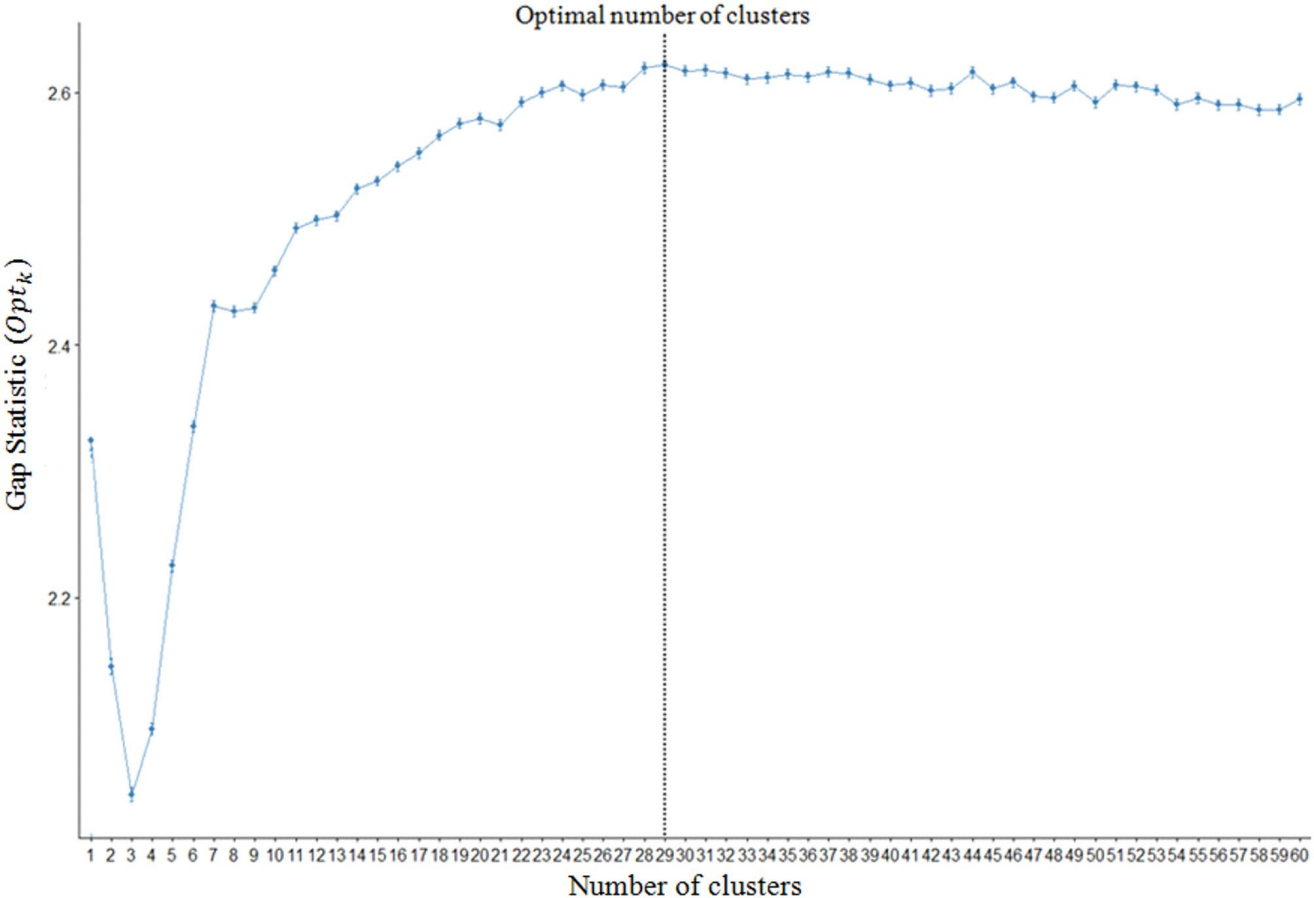
Specifying the optimum cluster number using GSM.

The determination of the optimum number of clusters presents a significant challenge in the K-MEANS algorithm. To address this issue, GSM is employed in order to identify the most appropriate number of clusters. As depicted in Fig. 8, the GSM yielded an optimum cluster number of$\:\:29$.

#### Comparative analysis of proposed methodology at ILF approach

The performance profile of the proposed methodology is validated using testing results of smart meters. The efficacy of the proposed KMEANS-NN strategy is assessed through a significant comparison with four widely used forecasting methods: ARIMA, CTREE, MLP and NNETAR. To evaluate the impact of KMEANS-NN on prediction accuracy, the performance of these forecasting methods with and without KMEANS-NN is precisely analyzed from $\:July\:{24}^{th},\:2023\:$to$\:\:December\:{31}^{st},\:2023$. The results that are documented in Table 2 averaged across all tested meters demonstrating that the incorporation of the KMEANS-NN significantly enhances the performance of all four forecasting methods and reduces the computational time in addition to improves prediction accuracy compared to the same methods applied individually to each meter. There is a significant performance improvement for the integration of MLP, NNETAR, CTREE and ARIMA strategies with the proposed KMEANS-NN as compared to the same four methods per individual meter. In this regard, the MAAPE for these four integrations is minimized by$\:\:22.21\%$, $\:25.62\%$, $\:12.72\%$ and $\:10.26\%$, respectively.

**Fig. 9:**
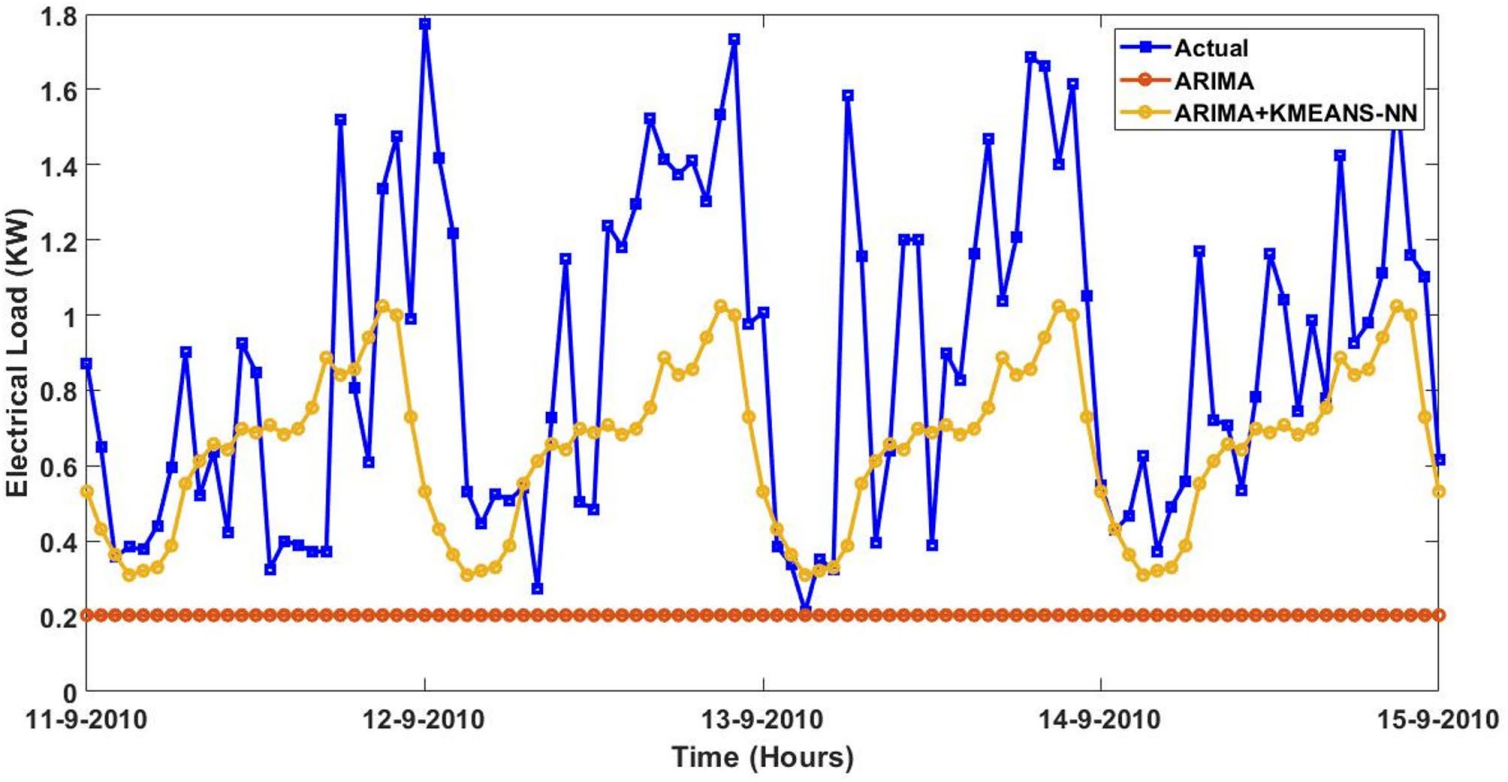
Predicted vs. actual electricity consumption profile of smart meter that is numbered at $\:1628$ using the ARIMA strategy with and without KMEANS-NN topology.

**Fig. 10:**
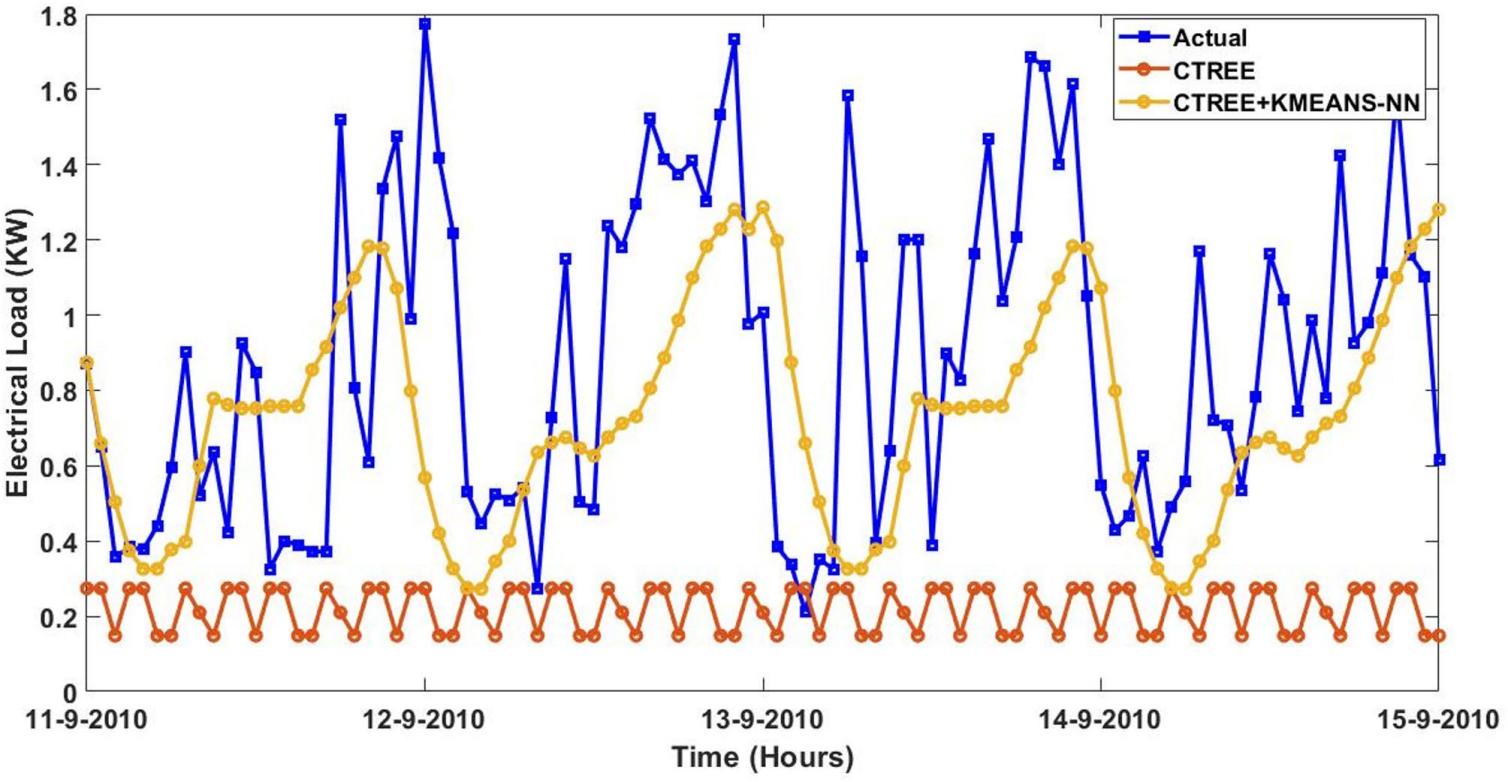
Predicted Vs. actual electricity consumption profile of smart meter that is numbered at $\:1628$ using the CTREE strategy with and without KMEANS-NN topology.

**Fig. 11:**
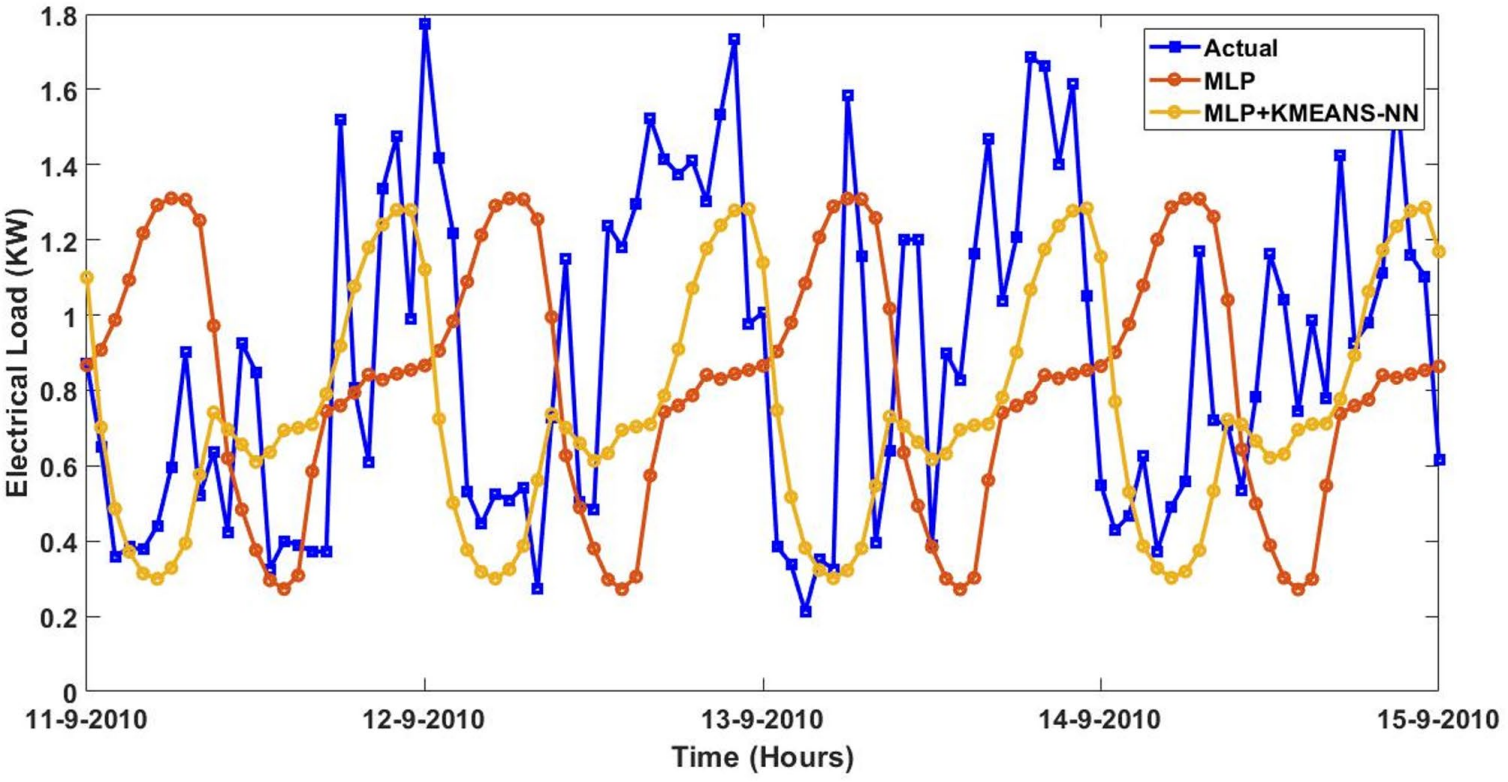
Predicted Vs. actual electricity consumption profile of smart meter that is numbered at $\:1628$ using the MLP strategy with and without KMEANS-NN topology.

**Fig. 12:**
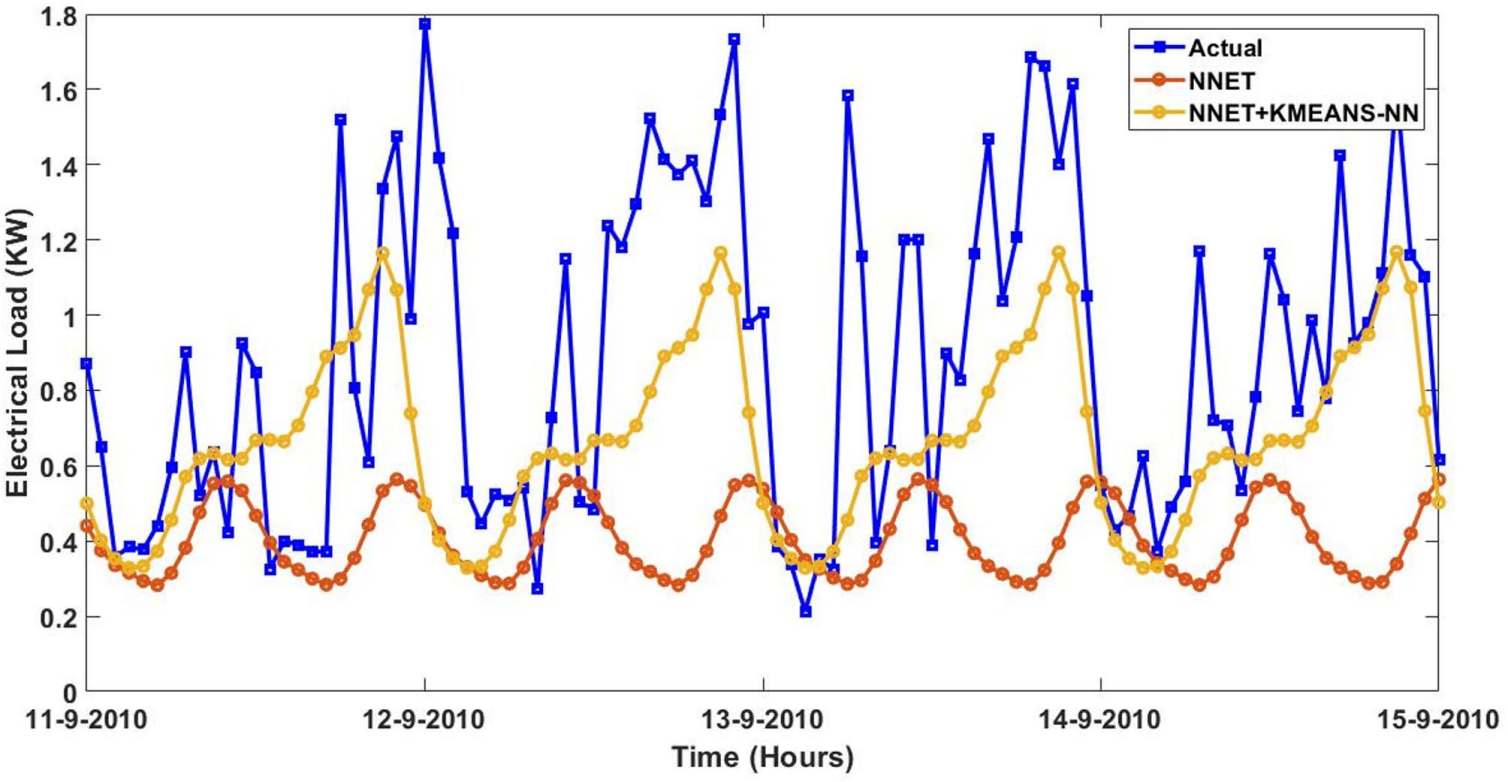
Predicted Vs. actual electricity consumption profile of smart meter that is numbered at $\:1628$ using the NNETAR strategy with and without KMEANS-NN topology.

Furthermore, the actual and predicted values by the four proposed forecasting methods on the four-days started from $\:August\:{11}^{th},\:2023\:$to$\:\:August\:{14}^{th},\:2023$ utilizing the trained data of smart meter that is numbered at $\:1628$ are expressed graphically with and without the integrated model of KMEANS-NN. The four forecasting methods with the proposed KMEANS-NN accurately predicts energy consumption, particularly during spikes and troughs. The proposed KMEANS-NN effectively captures the pattern of actual electricity consumption as demonstrated by the performance profile of the four-forecasting models diagrammed in a sequential pattern from Figs. 9, 10, 11 and 12.

**Table 2:** Comparative operational evaluation of the proposed LF methods.

Method	Methodology	Computational time	$\:RMSE$	$\:MAAPE$	$\:MAE$
MLP	Per individual meter	$\:03\:d\:16\:h\:12\:min$	$\:0.6201$	$\:0.5230$	$\:0.5140$
	With KMEANS-NN	$\:01\:h\:21\:min\:18\:s$	$\:0.5119$	$\:0.4073$	$\:0.4162$
NNETAR	Per individual meter	$\:03\:d\:23\:h\:09\:min$	$\:0.6168$	$\:0.5066$	$\:0.4959$
	With KMEANS-NN	$\:02\:h\:44\:min\:6\:s$	$\:0.4410$	$\:0.3768$	$\:0.3502$
CTREE	Per individual meter	$\:01\:d\:07\:h\:31\:min$	$\:0.5589$	$\:0.5006$	$\:0.4687$
	With KMEANS-NN	$\:31.57\:min$	$\:0.5175$	$\:0.4369$	$\:0.4266$
ARIMA	Per individual meter	$\:07\:d\:41\:h\:19\:min\:15\:s$	$\:0.5598$	$\:0.4178$	$\:0.4256$
	With KMEANS-NN	$\:02\:h\:33\:min\:42\:s$	$\:0.4589$	$\:0.3749$	$\:0.3598$

To comprehensively evaluate the performance of the proposed load forecasting methods, we incorporated six key performance indicators (KPIs): Mean Squared Relative Error (MSRE), Root Mean Squared Relative Error (RMSRE), Mean Absolute Error (MAE), Mean Absolute Relative Error (MARE), Mean Absolute Percentage Error (MAPE) and Root Mean Squared Percentage Error (RMSPE). These metrics were calculated for each forecasting method, both at the individual meter level and using the clustering-based approach. The results in Table 3 demonstrate that the proposed clustering-enhanced models consistently outperform their counterparts across all metrics, indicating improved accuracy and robustness.

Mean Squared Relative Error (MSRE)

31 $$\:MSRE=\:\frac{1}{n}\sum\:_{t=1}^{n}{\left(\frac{{A}_{t-}{F}_{t}}{{A}_{t}}\right)}^{2} \:$$.

Root Mean Squared Relative Error (RMSRE):

32 $$\:RMSRE=\:\sqrt{\frac{1}{n}\sum\:_{t=1}^{n}{\left(\frac{{A}_{t-}{F}_{t}}{{A}_{t}}\right)}^{2}}$$

33 $$\:RMSRE=\:\sqrt{\frac{1}{n}\sum\:_{t=1}^{n}{\left(\frac{{A}_{t-}{F}_{t}}{{A}_{t}}\right)}^{2}}$$

Mean Absolute Relative Error (MARE)

34 $$\:MARE=\:\frac{1}{n}\sum\:_{t=1}^{n}\left|\frac{{A}_{t-}{F}_{t}}{{A}_{t}}\right|$$.

Mean Absolute Percentage Error (MAPE):

35 $$\:MARE=\:\frac{100}{n}\sum\:_{t=1}^{n}\left|\frac{{A}_{t-}{F}_{t}}{{A}_{t}}\right|$$

Root Mean Squared Percentage Error (RMSPE):

36 $$\:\text{RMSPE}=\sqrt{\frac{{100}^{2}}{\text{n}}}\sum\:_{\text{t}=1}^{\text{n}}{\left(\frac{{\text{A}}_{\text{t}-}{\text{F}}_{\text{t}}}{{\text{A}}_{\text{t}}}\right)}^{2}$$

**Table 3:** Comparative evaluation of load forecasting models using multiple regression performance metrics.

Method	Methodology	MSRE	RMSRE	MARE	MAPE	RMSPE
MLP	Per individual meter	2.4144	1.3304	0.9107	91.0737	133.0389
	With KMEANS-NN	1.0218	0.8691	0.5836	58.3647	86.9089
NNETAR	Per individual meter	2.2418	1.286	0.8616	86.1626	128.5952
	With KMEANS-NN	0.6777	0.7206	0.4976	49.763	72.0644
CTREE	Per individual meter	1.9192	1.1952	0.8309	83.0867	119.5152
	With KMEANS-NN	1.1859	0.9468	0.6453	64.5298	94.6766
ARIMA	Per individual meter	0.8293	0.7451	0.5552	55.5219	74.5136
	With KMEANS-NN	0.6731	0.7204	0.4988	49.8769	72.0353

**Fig. 13:**
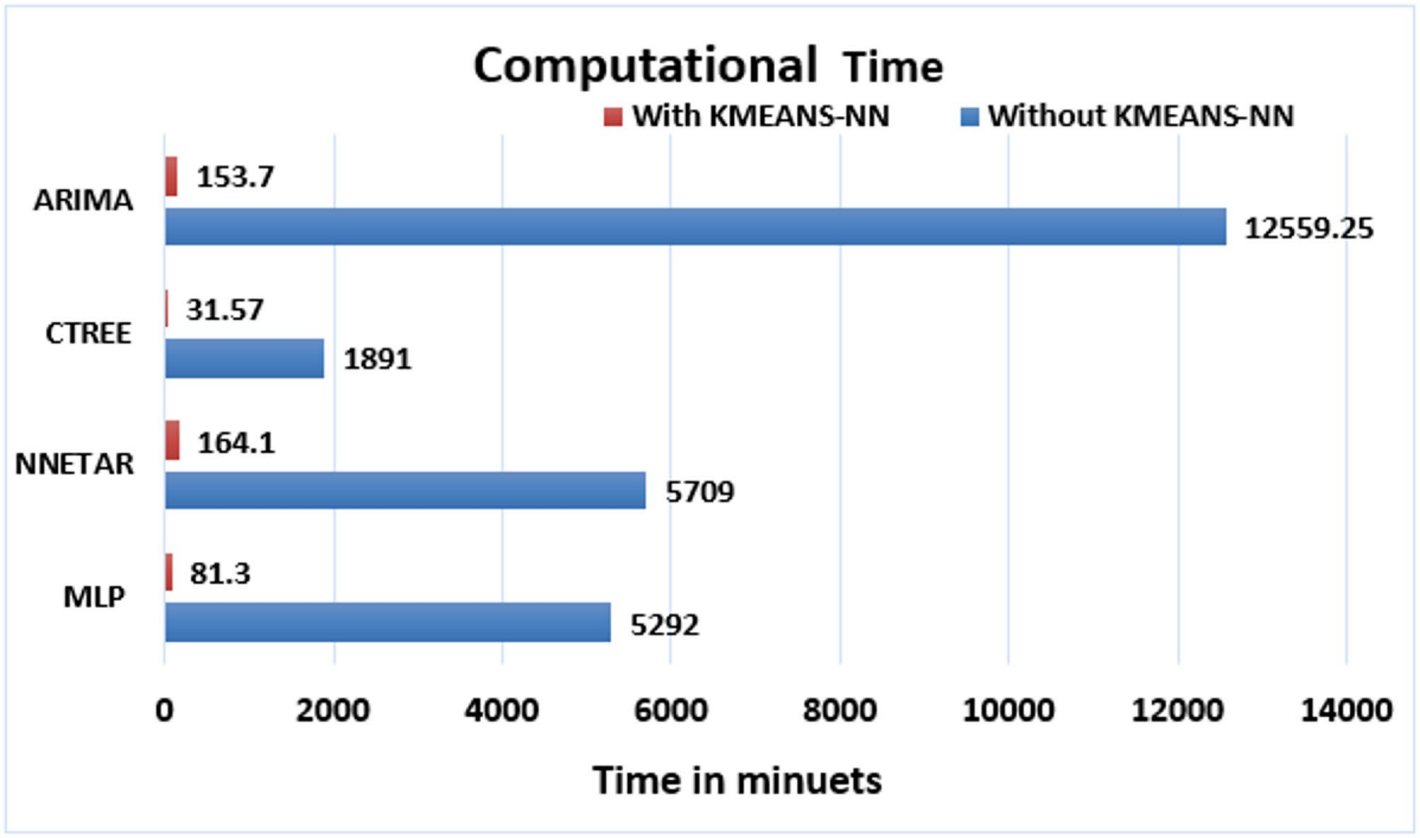
The computational time for the four forecasting methods with and without the proposed KMEANS-NN strategy.

Within the context of scientific measurements, the documented computational time that is expressed in Table 2 are shaped using standardized symbols to denote specific time units: $\:{\prime\:}s{\prime\:}$ for seconds, $\:{\prime\:}min{\prime\:}$ for minutes, $\:{\prime\:}h{\prime\:}$ for hours and $\:{\prime\:}d{\prime\:}$ for days. The computational efficiency of the four proposed forecasting methods presented in Table 2 merits careful consideration. As shown in Fig. 13, the computational time required for LF using the proposed KMEANS-NN strategy has been substantially reduced enabling the prediction of $\:2089$ individual smart meters to be completed within a few minutes or hours. This remarkable improvement in computational efficiency demonstrates the practical feasibility of the KMEANS-NN approach for real-world LF applications involving large numbers of smart meters. On the contrast, the computational time of the four forecasting methods applied on each meter individually become very excessive taking several hours to few days in order to complete prediction task.

**Fig. 14:**
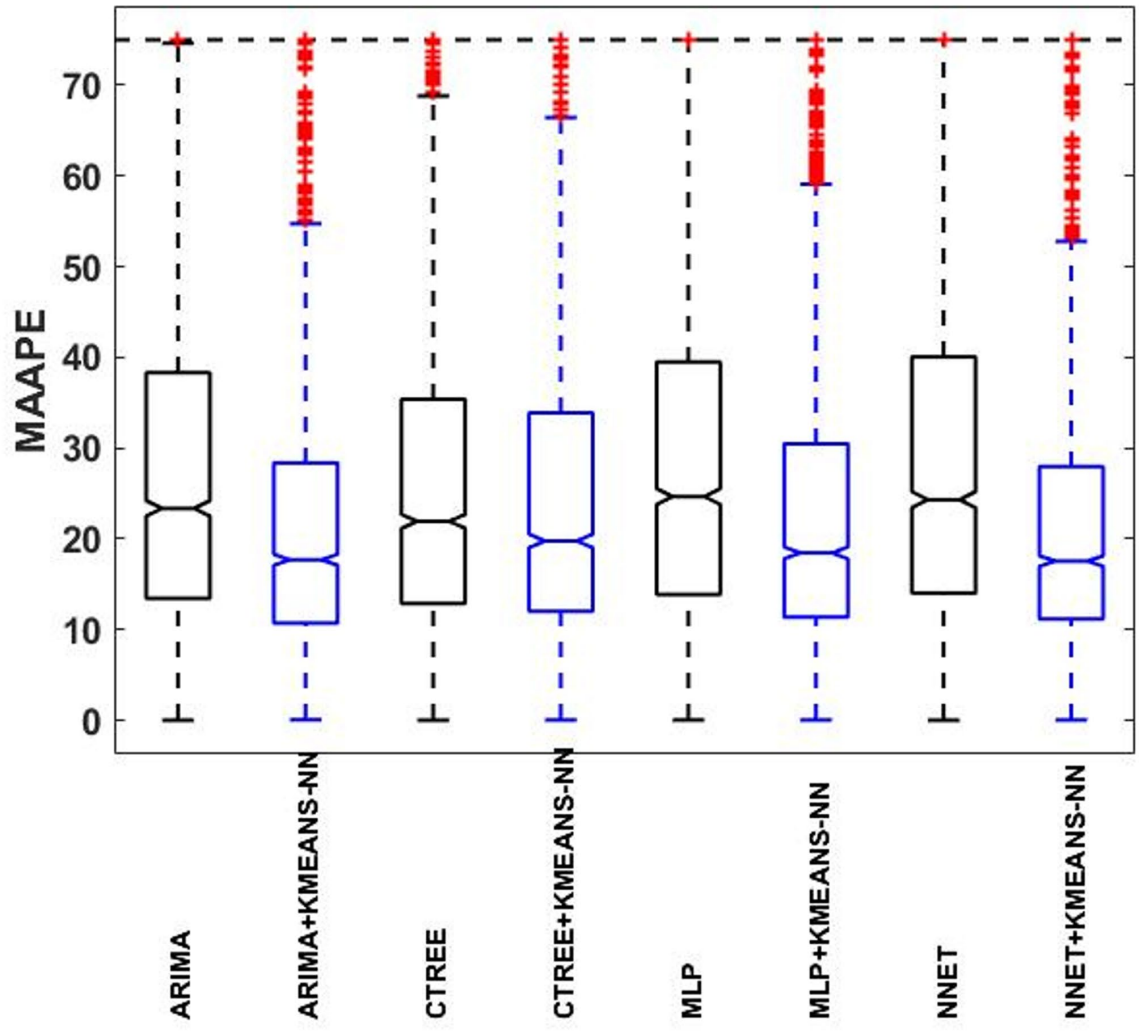
The box plot of the $\:RMSE$ for all meter’s result from four forecasting methods with and without the proposed KMEANS-NN strategy.

**Fig. 15:**
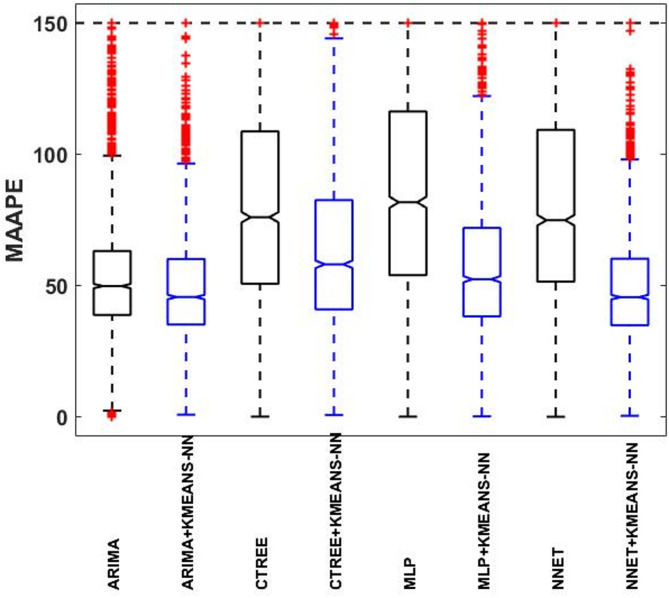
The box plot of the $\:MAAPE$ for all meter’s result from four forecasting methods with and without the proposed KMEANS-NN strategy.

**Fig. 16:**
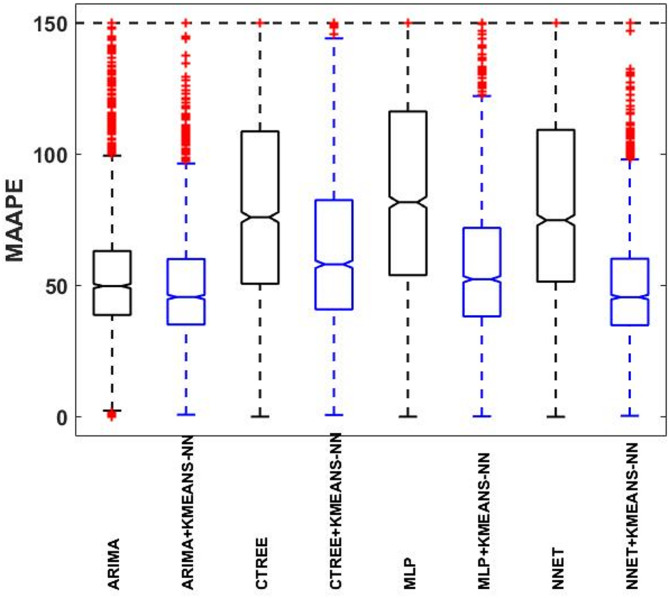
The box plot of the $\:MAE$ for all meter’s result from four forecasting methods with and without the proposed KMEANS-NN strategy.

**Fig. 17:**
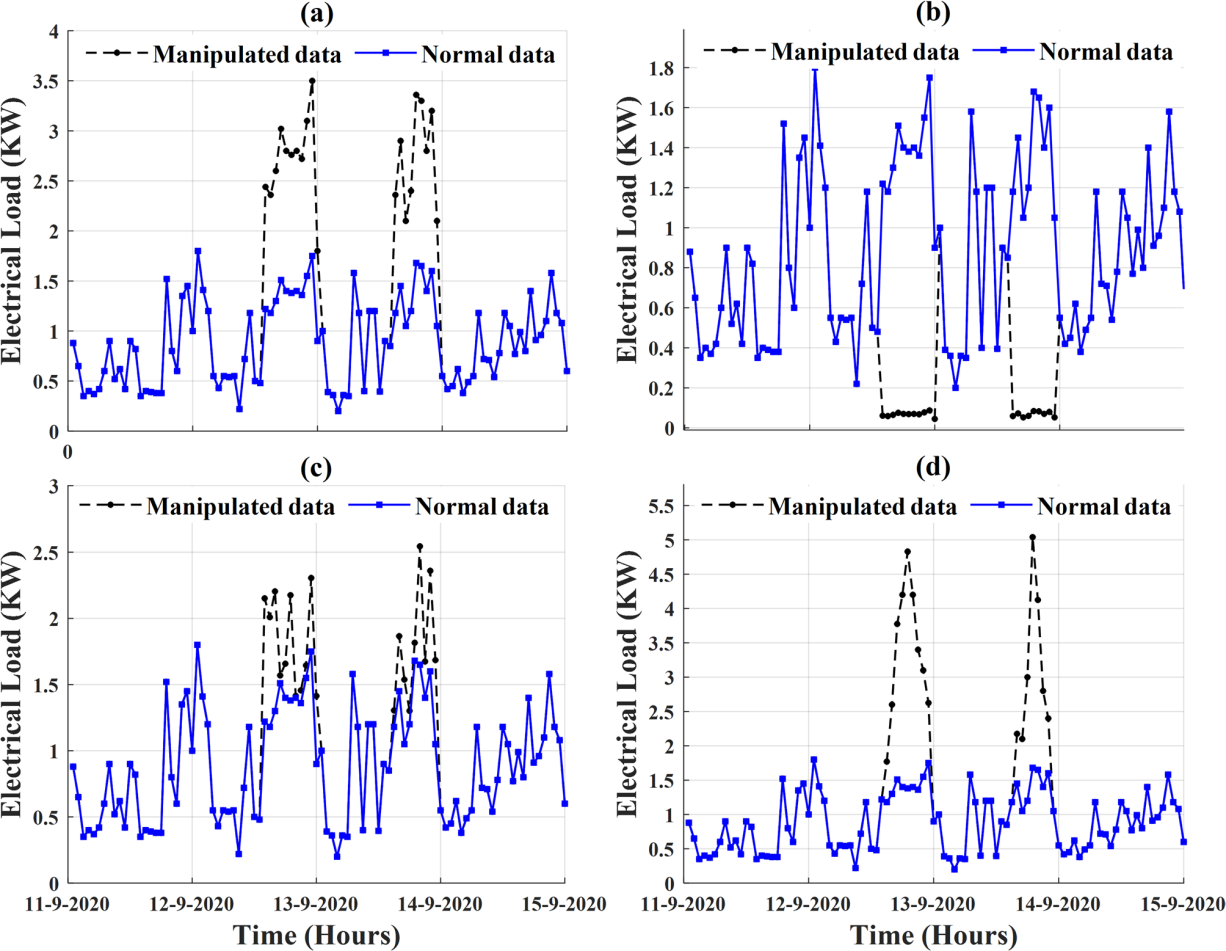
A sample of electricity consumption data of smart meter that is numbered at $\:1628$ with and without different cyber-attack scenarios; (**a**) $\:{1}^{st}\:$scaling attacks, (**b**) $\:{2}^{nd}\:$scaling attacks, (**c**) random attacks and (**d**) ramping attacks.

Also, the box plot is used for comparing the error at individual meters on the four proposed forecasting methods with and without the usage of KMEANS-NN strategy. Box plot is a graphical representation that shows important statistical profile including the median, the first quartile and the third quartile of the trained data (represents $\:25\%$ and $\:75\%$ from the trained data respectively). The sequential box plots that start from Figs. 14, 15, 16 and 17 highlight the operational impact of MAE, MAAPE and RMSE of each method individually compared to the proposed KMEANS-NN strategy. Also, the statistical error results of this study with and without the application of KMEANS-NN strategy is documented in Table 3. The diagrammed box plots emphasized the significant capability of the proposed KMEAN-NN strategy to improve the overall error statistics of MAE, MAAPE and RMSE for the four forecasting methods. The discussed results in Table 4 have propped that KMEAN-NN approach reduced the median, the first and the third quartile value of the prediction errors for the four proposed forecasting methods compared to the forecasting methods for each meter separately without the proposed KMEAN-NN technique.

**Table 4:** Statistical error analysis for the four forecasting methods with$\:/$without KMEANS-NN strategy. individually refers to (Per individual Meter) proposed refers to (KMEANS-NN Strategy).

Methodology	$\:RMSE$			$\:MAAPE$			$\:MAE$		
	$\:{1}^{th}Q$	median	$\:{3}^{rd}Q$	$\:{1}^{th}Q$	median	$\:{3}^{rd}Q$	$\:{1}^{th}Q$	median	$\:{3}^{rd}Q$
ARIMA strategy									
Individually	$\:13.44$	$\:23.34$	$\:38.32$	$\:38.85$	$\:49.82$	$\:63.20$	$\:0.16$	$\:0.29$	$\:0.47$
Proposed	$\:10.72$	$\:17.70$	$\:28.35$	$\:35.20$	$\:45.65$	$\:60.14$	$\:0.13$	$\:0.22$	$\:0.35$
CTREE strategy									
Individually	$\:12.88$	$\:21.91$	$\:35.38$	$\:50.73$	$\:76.04$	$\:108.7$	$\:0.18$	$\:0.30$	$\:0.49$
Proposed	$\:12.02$	$\:19.78$	$\:33.87$	$\:40.96$	$\:58.11$	$\:82.59$	$\:0.15$	$\:0.25$	$\:0.45$
MLP strategy									
Individually	$\:13.87$	$\:24.64$	$\:39.48$	$\:53.97$	$\:81.74$	$\:116.3$	$\:0.18$	$\:0.34$	$\:0.54$
Proposed	$\:11.38$	$\:18.46$	$\:30.47$	$\:38.27$	$\:52.44$	$\:71.97$	$\:0.14$	$\:0.23$	$\:0.38$
NNETAR strategy									
Individually	$\:14.03$	$\:24.30$	$\:40.05$	$\:51.54$	$\:74.90$	$\:109.3$	$\:0.18$	$\:0.31$	$\:0.54$
Proposed	$\:11.17$	$\:17.56$	$\:27.93$	$\:34.91$	$\:45.60$	$\:60.25$	$\:0.14$	$\:0.22$	$\:0.35$

#### Performance evaluation of proposed ADS under different cyber-attack scenarios

In this subsection, the performance profile of the proposed ADS under different cyber-attacks scenarios is evaluated. To achieve this step, $\:50\%$ of the collected dataset is manipulated with the different types of cyber-attack scenarios$\:/$models. Figure 14 presents a sample of the electricity consumption data of the smart meter that is numbered at $\:1628$ with different types of cyber-attack scenarios (anomalous data). The first two scenarios are defined as scaling attacks; the first scenario aims to increase the level of electricity consumption data during different hours on different days whereas the second scenario aims to reduce the level of energy consumption data during different hours on different days. The next two attack scenarios are random and ramping attacks, respectively. As shown in Fig. 14(c), random attacks pose a challenge to the proposed ADS as their behaviors/patterns are very similar to patterns of electricity consumption data.

**Fig. 18:**
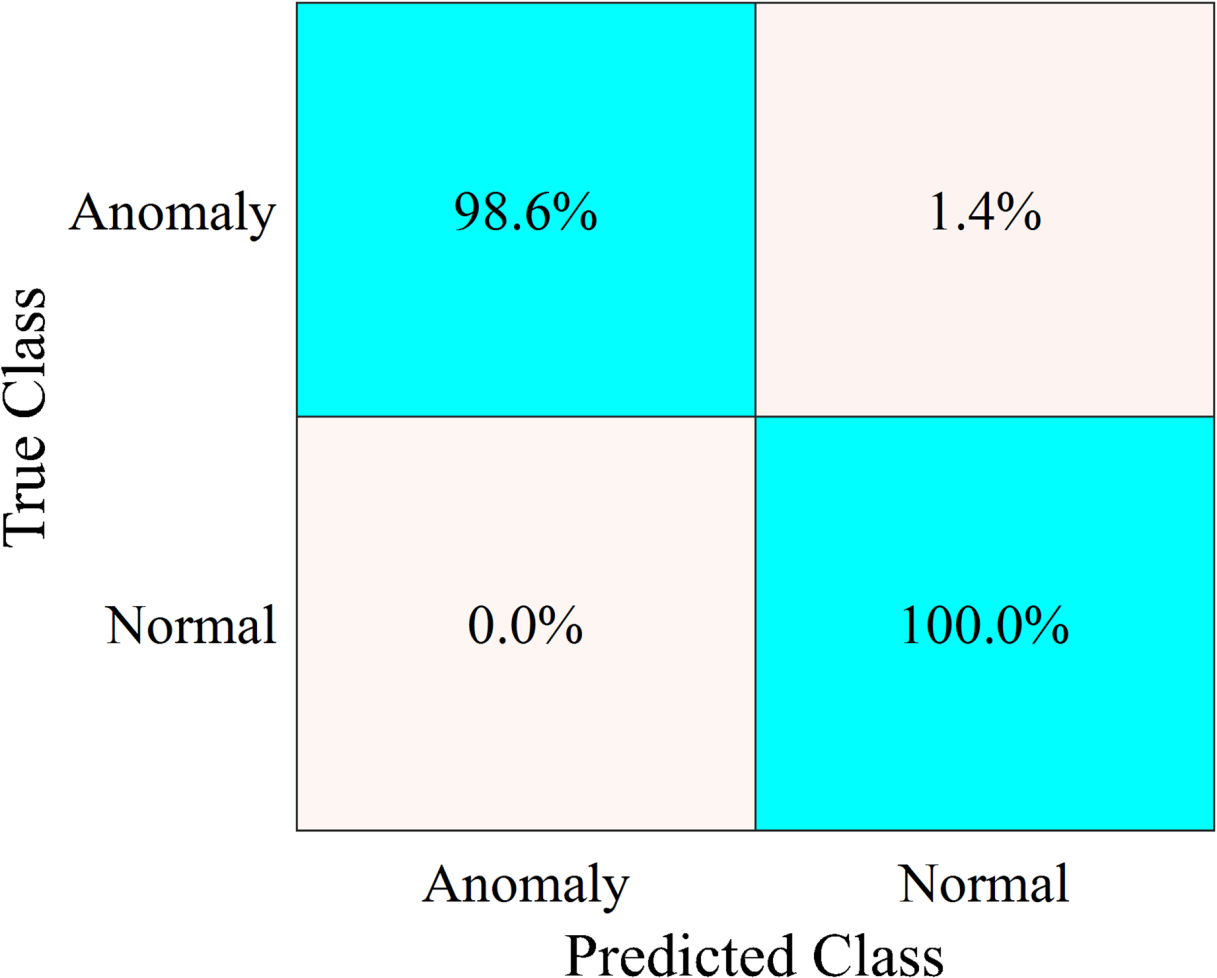
Confusion matrix of the classification results for the manipulated dataset of smart meter that is numbered at $\:1628$.

After adding different cyber-attack scenarios to the trained dataset, the specific features of the dataset are extracted and calculated. Sequentially, these features are transformed by PCA. The transformed features are fed into the trained OCSVM model to identify the anomalous data and their corresponding attack meters. Deeping on the effectiveness of ADS strategy in enhancing the ILF profile, Fig. 18 addresses the classification results for the dataset of smart meter that is numbered at $\:1628$ after adding different types of cyber-attack scenarios to $\:50\%$ of its dataset.

The performance of the proposed ADS is not affected by the added anomalous data to classify normal data samples where the classification accuracy of the normal data samples is still$\:\:100\%$. For the anomalous data samples induced by cyber-attack scenarios, the trained OCSVM model successfully identified $\:98.6\%$ of manipulated$\:/$anomalous data samples. The remaining $\:1.4\%$ of the trained data is confused with the normal data samples whose patterns resemble anomalous data samples resulting from low-amplitude smooth random attacks.

**Fig. 19:**
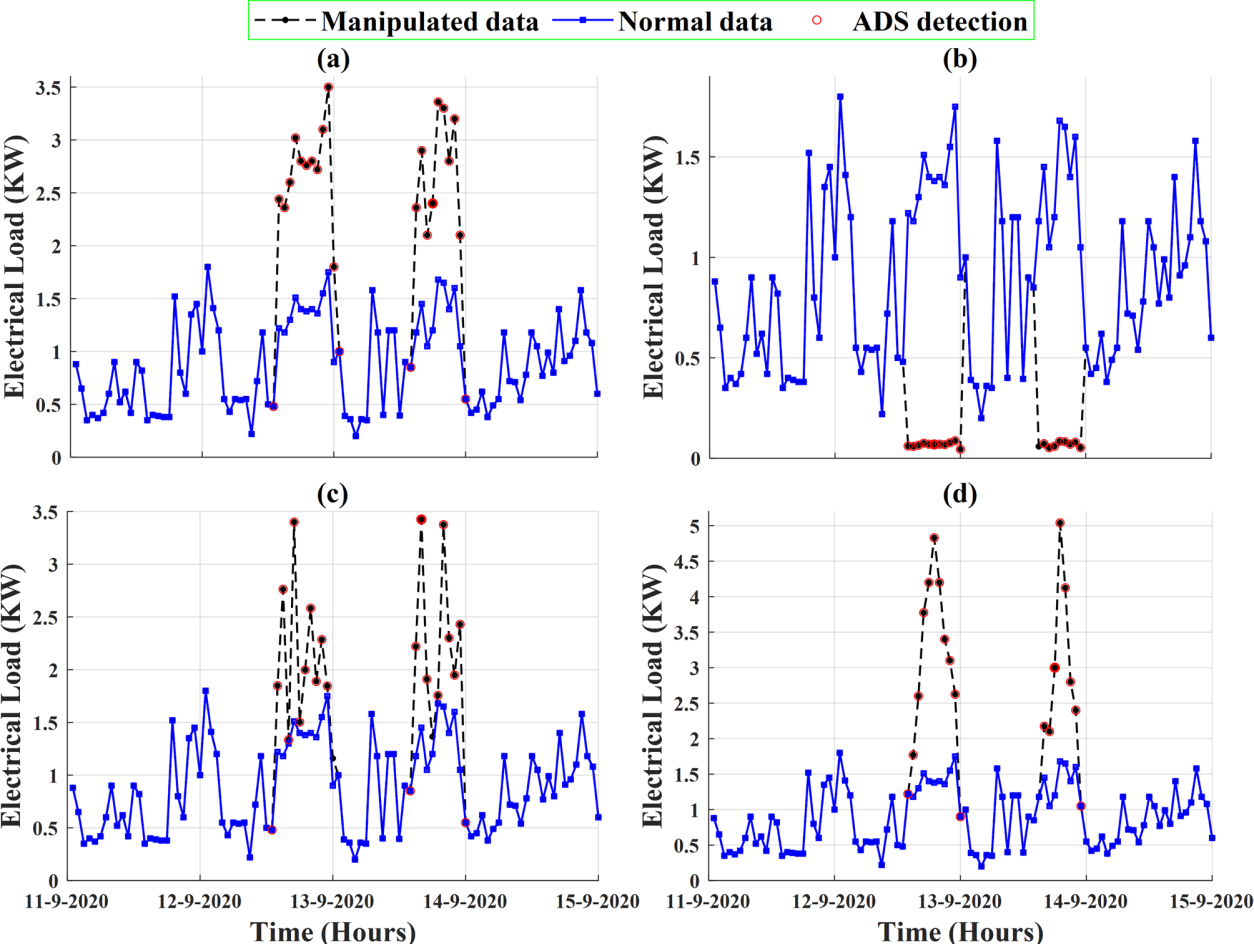
Anomaly data detection using the proposed ADS for a sample of electricity consumption data of smart meters that is numbered at $\:1628$ with different attacks; (**a**) $\:{1}^{st}\:$scaling attacks, (**b**) $\:{2}^{nd}\:$scaling attacks, (**c**) random attacks and (**d**) ramping attacks.

The overall evaluation matrices representing the performance indices of accuracy, sensitivity, precision, specificity and F1-score of the proposed ADS are appropriately obtained at$\:\:99.3\%$,$\:\:100\%$,$\:\:98.62\%$, $\:98.6\%\:$and $\:0.9896$ respectively. Furthermore, Fig. 19 shows the manipulated/anomalous data detection using the proposed ADS for a sample of electricity consumption data of smart meters that is numbered at $\:1628$ with anomalous data resulting from the different proposed types of cyber-attack scenarios. So, the performance results of this study demonstrated the superiority and effectiveness of the proposed ADS in detecting anomalies in smart meter telemetry data which subsequently affect the performance and results of ILF approach.‎ The proposed ADS is an important tool for system operators to be aware of which smart meters have been attacked and then take remedial measures to deal with the detected anomalous data and secure smart meter telemetry data against future cyber-attacks. For advanced studies, remedial measures against cyber-attacks are a matter of future work and predominant challenges. The ADS results emphasized the powerful impact of this strategy in overcoming hazardous hacking actions and increasing the stability degree of the forecasting profile in making precise predictions with a reasonable efficiency.

**Fig. 20:**
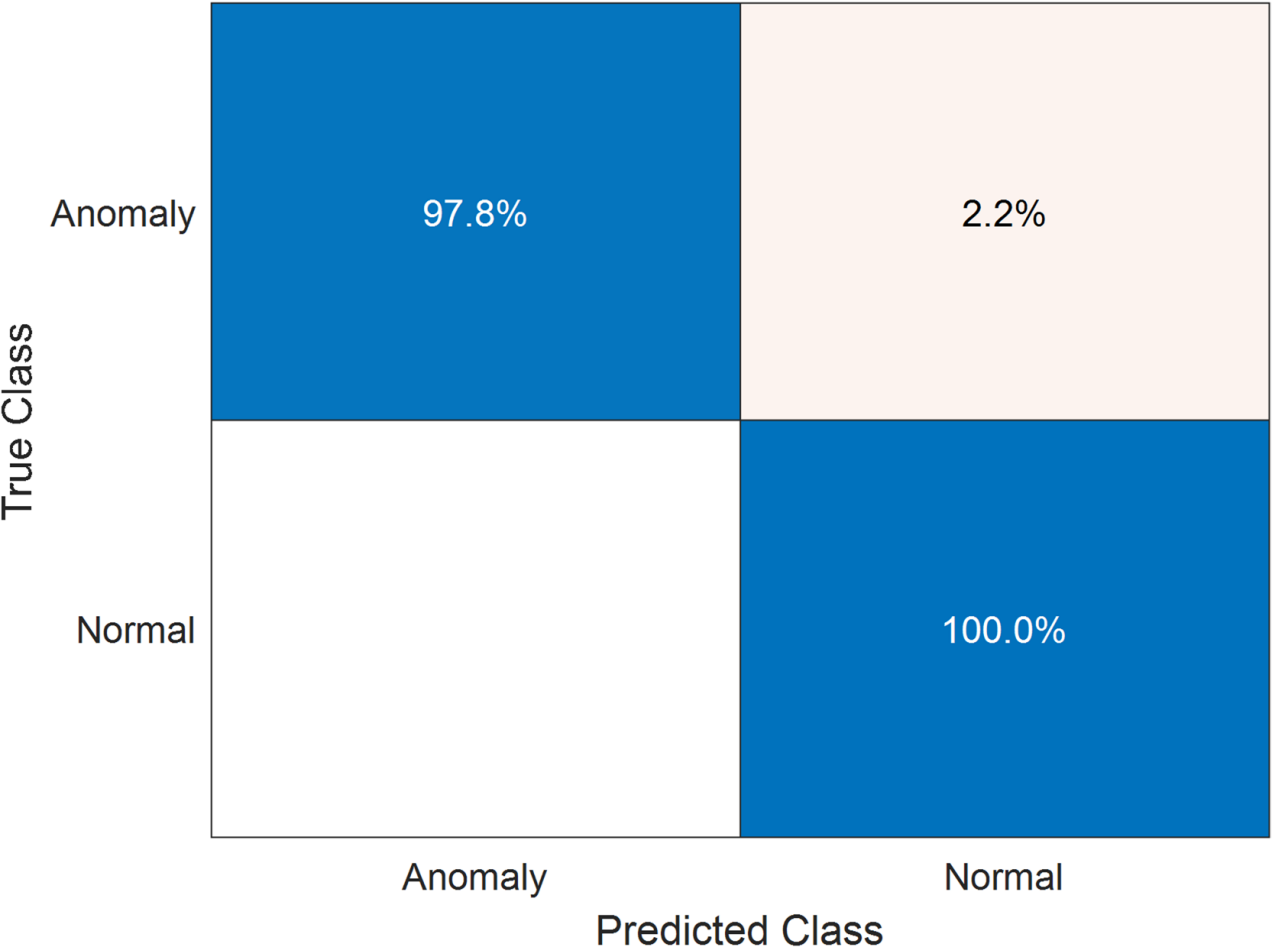
Confusion matrix of the classification results for the manipulated dataset of smart meter that is numbered at $\:1628$, when combining all attack models.

**Fig. 21:**
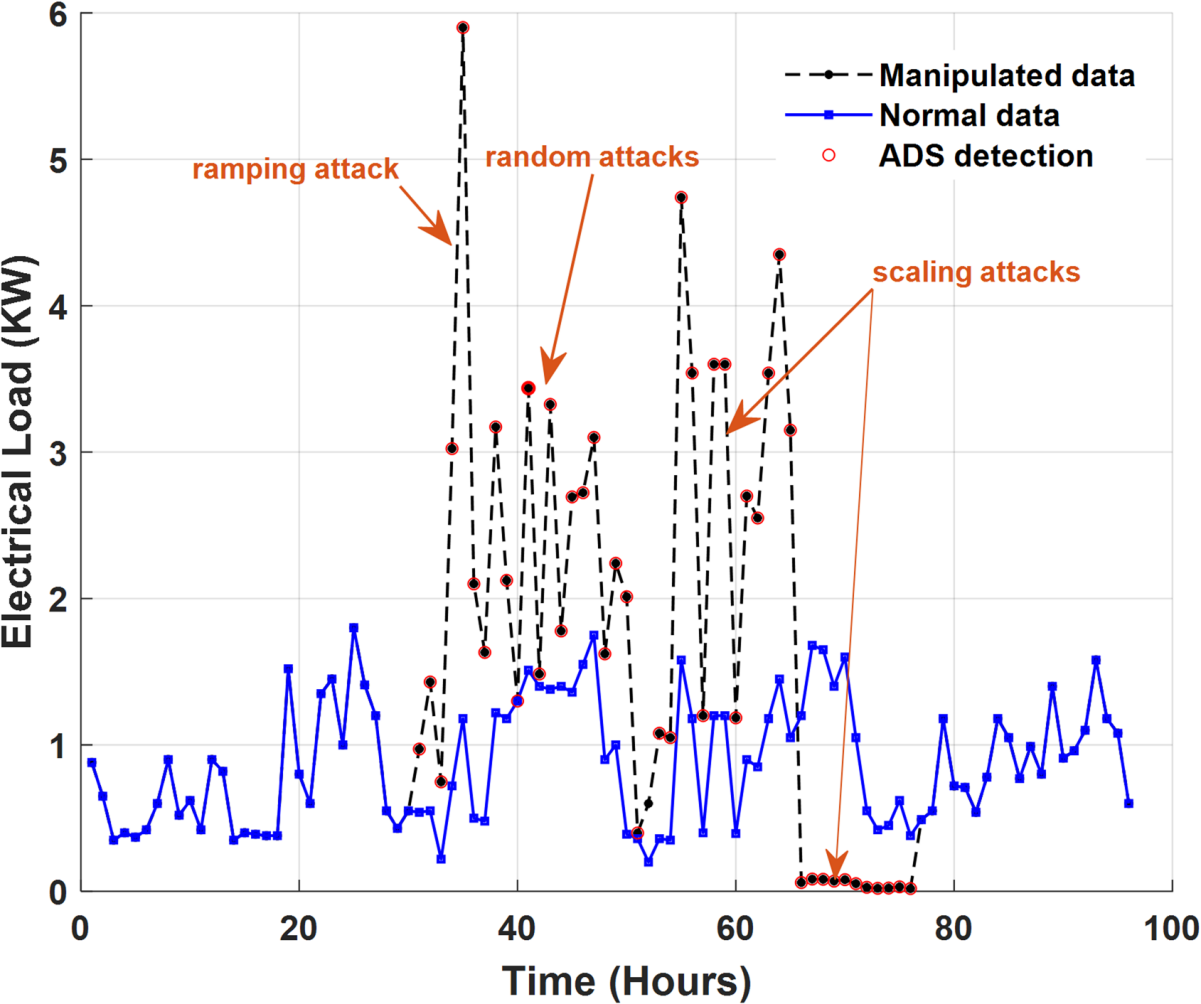
Anomaly data detection using the proposed ADS for a sample of electricity consumption data of smart meters that is numbered at $\:1628$, when combining all attack models.

The performance of the proposed ADS is only marginally affected when all attack models are injected/combined into normal data samples, as classification accuracy for normal data remains at$\:\:100\%$. For anomalous data samples generated by all cyberattack models, the trained OCSVM model successfully detected $\:97.8\%$ of the manipulated/anomalous samples. The remaining $\:2.2\%$ were misclassified as normal samples due to their similarity to low-amplitude, smooth random attacks. Figure 20 in manuscript illustrates the classification results for the smart meter dataset (No. 1628) after combining all cyber-attack models within its data. So, the overall evaluation metrics of accuracy, precision, recall, and F1-score for the proposed ADS, when all attack models are combined, are $\:98.9\%,$ $\:100\%,\:97.8\%$, and $\:0.989$, respectively, demonstrating the high reliability and robustness of the system. Furthermore, Fig. 21 in paper illustrates detection of manipulated/anomalous data using the proposed ADS for a sample of electricity consumption data from the smart meter No.1628, with anomalies resulting from the combination of all cyber-attack models. Therefore, the performance results highlight the capability of the proposed ADS to detect anomalies in smart meter telemetry data, even under simultaneous and complex multi-model cyber-attacks.

## Conclusions

This study addressed the critical challenges of computational inefficiency and cybersecurity in individual smart meter electricity demand forecasting. A new strategy was proposed which integrated K-MEANS clustering alongside Neural Networks to optimize the performance of the forecast in terms of precision and speed. Smart meters were grouped based on similarity in consumption behavior using K-MEANS clustering, and then cluster-level average profiles were used to train neural network models. These models were further refined using the actual readings of individual meters to preserve accuracy at the meter level while avoiding the burden of training separate models for each device.

To enhance system resilience against cyber-attacks, the suggested framework included a PCA and OCSVM based ADS. The ADS detected anomalous patterns and false data injection attacks in the telemetry data for ensuring the integrity of the forecasting procedure. The approach was tested with five months of hourly electricity consumption data gathered from 2,089 smart meters. It showed excellent forecasting accuracy, anomaly detection, and scalability for deployment in real-world smart grid systems. The key findings can be summarized in the following points:

1. Compared to classical individual forecasting methods, KMEANS-NN, as proposed, reduced MAAPE by as much as 25.6% while also showing consistent improvements in MAE and RMSE.
2. The model made predictions for over 2,000 smart meters within minutes, compared to several days by per-meter training methods. This made the model suitable for large-scale and real-time prediction applications.
3. The integrated ADS managed to identify 98.6% of tampered data samples generated from cyber-attack patterns such as scaling, ramping, and random attacks. It achieved 99.3% classification accuracy, 100% sensitivity, 98.62% precision, and 98.6% specificity, with 100% accuracy on clean data samples.
4. When used in conjunction with ARIMA, CTREE, MLP, and NNETAR models, the suggested framework improved prediction performance consistently, validating its applicability for various algorithmic architectures.
5. Their approach was tested against the electricity consumption data from 2,089 smart meters over the course of five months. The results confirmed its reliability and adaptability with regard to different consumption patterns and numerous load profiles.
6. The integration of clustering, forecasting, and anomaly detection into one cohesive framework improved operational reliability, and cybersecurity. This holistic approach guaranteed accurate and reliable lLF, aiding utility planning and strengthening the power grid’s stability, accuracy and dependability.

In this context, the economics of the model is related the efficiency gain and market balance due to the higher scored accuracy and low calculated error of performance indices. Future work will try to enhance the suggested methodology by incorporating exogenous variables like climatic conditions, i.e., wind speed, temperature, humidity, and rainfall. These variables affect the consumption of electricity and should further increase the predictive power and generality of the model. Further, the developed framework will be implemented on data of different regions and customer categories such as residential, commercial, and industrial. This will challenge its robustness and scalability under different operating conditions. Future research includes implementing methodology on different prediction horizons and levels of aggregation and ascertaining its viability integration with real-time demand response and grid management systems.

## Data Availability

All data generated or analysed during this study are included in this published article.

## Abbreviations

P2P: Peer-to-Peer
OCSVM: One-class support vector machine
PCA: Principal component analysis
LF: Load forecasting
RES: Renewable energy sources
PV: Photo-voltaic
IESs: Integrated energy systems
SGC: State grid corporation
ILF: Individual load forecasting
ALLF: Aggregated-level load forecasting
LRM: Linear regression method
SVR: Support vector regression
ANNs: Artificial neural networks
ARIMA: Auto regressive integrated moving average
DL: Deep learning
ML: Machine learning
RNNS: Recurrent neural networks
LSTM: Long short-term memory
GRU: Gated recurrent units
STLF: Short-term load forecasting
TMB: Temporal mixer block
DLA: Deep learning analysis
IOT: Internet of things
NN: Neural network
EDS: Electrical distribution sector
SCA: Suez Canal Authority
ADS: Anomaly detection scheme
MAAPE: Mean arctangent absolute percentage error
CTREE: Conditional inference trees
MLP: Multi-layer perceptron
MICE: Multivariate imputation chained equations
NNETAR: Neural network autoregressive
GSM: Gap statistic method
RMSPE: Root mean squared percentage error
RMSRE: Root mean squared relative error
DFD: Diagnostic feature designer
MAE: Mean absolute error
KPIs: Key performance indicators
${\varvec{MSRE}}$: Mean squared relative error
${\varvec{RMSE}}$: Root mean square error
${\varvec{MAPE}}$: Mean absolute percentage error
WCSS: Within-cluster sum of squares
${\varvec{x}}_{{{\varvec{ij}}}}$: Energy consumption coefficient
${\varvec{i}}^{{{\varvec{th}}}}$: Given labeled smart meter
${\varvec{j}}^{{{\varvec{th}}}}$: Given labeled time step
$${\varvec{k}}$$: Weekdays operator
${\varvec{n}}$: Total number of hourly days
${\varvec{w}}$: Total number weekdays
${\varvec{h}}$: Hourly day operator
$${\varvec{x}}_{{\varvec{i}}}$$: Observation operator
$${\varvec{x}}$$: Sample data
${\varvec{x}}_{{{\varvec{i}},{\varvec{t}}}}^{{\varvec{a}}}$: Attack $/$ manipulated telemetry data
${\varvec{x}}_{{{\varvec{i}},{\varvec{t}}}}$: Original $/$ clean telemetry data
${\varvec{k}}_{{\varvec{s}}}$: Scaling attack operator
${\varvec{t}}_{{\varvec{i}}}$: Lower scaling attack time limit
${\varvec{t}}_{{\varvec{e}}}$: Upper scaling attack time limit
${\varvec{k}}_{{\varvec{r}}}$: Ramping attack coefficient
${\varvec{k}}_{{\varvec{d}}}$: Scale parameter of random attack
${\varvec{R}}_{{\varvec{t}}}$: Positive normalization operator
${\mathbf{\mathcal{M}}},$: Mean error coefficient
${\varvec{\rho}},{\varvec{\omega}}$: Hyper-plane parameters
$${\varvec{\xi}}_{{\varvec{i}}}$$: Slack variable
$${\varvec{\psi}}$$: Classifying operator
${\varvec{A}}_{{\varvec{t}}}$: Actual meter reading value
${\varvec{F}}_{{\varvec{t}}}$: Forecasted load output
${\varvec{nn}}$: Total number of reading samples
${\varvec{TP}}$: Correctly classified normal data
${\varvec{TN}}$: Detected anomalous data
${\varvec{FN}}$: Number of normal data samples
${\varvec{FP}}$: Anomalous data samples
